# Symptom burden in multiple long-term conditions: An AI-supported, mixed-methods concept elicitation study

**DOI:** 10.1177/20542704261459717

**Published:** 2026-07-15

**Authors:** Sarah E Hughes, Benjamin M A Hughes, Shamil Haroon, Eleanor Hathaway, Nicola Anderson, Christel McMullan, Michael Newnham, Clare Taylor, Alastair Denniston, Steven Backhouse, Andrew Filer, Karim Raza, Julia Scarisbrick, Clare Anderson, Matthew Lee, Lee Watts, Victoria Hodgetts Morton, Sarah Hillman, Faraz Mughal, Ed Day, Tariq Sami, Aliza Jesani, Thomas Jackson, Philip Collis, John Devin Peipert, Melanie Calvert

**Affiliations:** 1 Centre for Patient Reported Outcome Research, 1724 University of Birmingham, Birmingham, Birmingham, UK; 2 Department of Applied Health Sciences, College of Medicine and Health, University of Birmingham, UK; 3 National Institute of Health and Care Research (NIHR) Blood and Transplant Research Unit in Precision Cellular Therapeutics, University of Birmingham, Birmingham, UK; 4 NIHR Applied Research Collaboration West Midlands, University of Birmingham, Birmingham, UK; 5 NIHR Biomedical Research Centre, University of Birmingham, Birmingham, UK; 6 University Hospitals Birmingham NHS Foundation Trust, Birmingham, UK; 7 97599 Cwm Taf Morgannwg University Health Board, Princess of Wales Hospital, Bridgend, UK; 8 Department of Inflammation and Ageing, University of Birmingham, Birmingham, UK; 9 Department of Immunology and Immunotherapy, University of Birmingham, Birmingham, UK; 10 Centre for Human Brain Health, School of Psychology, 1724 University of Birmingham, Birmingham, UK; 11 School of Medicine, Keele University, Keele, Staffordshire, UK; 12 Oxford Primary Care Clinical Trials Unit, Nuffield Department of Primary Care Health Sciences, University of Oxford, Oxford, UK; 13 Institute for Mental Health, School of Psychology, 1724 University of Birmingham, Birmingham, UK; 14 Birmingham and Solihull Mental Health Foundation Trust, Birmingham, UK; 15 1731 Sandwell and West Birmingham Hospitals (NHS) Trust, Birmingham, West Midlands, UK; 16 8238 Walsall Healthcare NHS Trust, Walsall, Birmingham, UK; 17 Patient Partner, Centre for Patient Reported Outcome Research, University of Birmingham, Birmingham, UK

**Keywords:** concept elicitation, patient-reported outcome, PRO, PROM, AI, large language models, mixed-methods, multi-morbidity, multiple long-term conditions, content validity

## Abstract

**Objective:**

To develop the conceptual framework for an MLTC-specific patient-reported outcome measure (PROM), the Symptom Burden Questionnaire™ for MLTC (SBQ™-MLTC).

**Design:**

Mixed-methods study: (1) symptom list generation; (2) assessment of list face validity; and (3) construction of a conceptual framework.

**Setting:**

Concept elicitation and conceptual framework development using existing PROMs identified through the Mapi Research Trust PROQOLID eCOA database and AI-generated symptom lists.

**Participants:**

Fifty-one condition-specific PROMs with evidence of patient involvement during concept elicitation were included for symptom extraction. ChatGPT-4 generated symptom lists for 24 conditions prevalent in MLTC. Seventeen healthcare practitioners reviewed symptom relevance and contributed to refinement of the conceptual framework.

**Main outcome measures:**

Identification, refinement, and organisation of relevant MLTC symptoms into body system and functional domains, and development of the SBQ™-MLTC's conceptual framework.

**Results:**

ePROVIDE searches in July and August 2023 identified 51 condition-specific PROMs for 24 conditions prevalent in MLTC. ChatGPT-4 was prompted to generate a list of 75 symptoms for each condition. A merged list of 2202 symptoms was iteratively reduced to 190 symptoms for healthcare practitioner review. The final conceptual framework included 151 symptoms spanning 18 body system and functional domains.

**Conclusions:**

This study represents the first phase in the development of an MLTC-specific PROM of symptom burden. Generative AI output triangulated with content from existing PROMs and healthcare practitioner review proved a feasible approach to concept elicitation. Planned cognitive debriefing will confirm content validity of the SBQ™-MLTC for people with lived experience. In the future, the SBQ™-MLTC could support integrated, symptom-led approaches for clinical management of MLTC.

## Introduction

Multiple long-term conditions (MLTC), or multimorbidity, is typically defined as the co-occurrence of two or more conditions within the same individual and is a recognised proxy for medical complexity.^1^ With an ageing global population, MLTC prevalence is rising and affects an estimated 37.2% of the adult population.^2^ Risk factors for MLTC include increasing age, female sex, and lower socioeconomic status. MLTC is associated with reduced quality of life, lower functional status, increased healthcare utilisation and increased mortality.^1,3^

Individuals living with MLTC experience increased symptom burden, which negatively impacts quality of life. Due to the large number of conditions prevalent in MLTC, experienced symptoms are heterogeneous, interrelated and may fluctuate..^4–7^ Symptom burden is a key outcome in the treatment of MLTC, where the focus of care is on disease management and symptom control.^7^ Symptoms may be a reflection of disease progression or a sequelae of treatment, and they may alter the trajectory of disease, leading to the development of new conditions or adverse events.^8^ High symptom burden is associated with poorer functional status^9^ and symptoms have been found to contribute to patients’ perceptions and understanding of overall health, more so than the underlying disease.^10^ Interpretation and management of symptoms are therefore central to the care of patients with MLTC; yet there has been limited methodological research into the measurement of symptom burden. To date, studies have primarily focused on measuring functioning, quality of life, treatment burden and care experience.^11,12^

Symptom-focused research in MLTC is challenging due to the complex interactions between multiple diseases, treatments and a siloed medical model that focuses on individual diseases.^13^ The UK's National Institute for Health and Care Excellence (NICE) recommends a holistic, person-centred approach to MLTC management that is responsive to patient goals and preferences, aiming to improve quality of life by reducing treatment burden, adverse events and unplanned or uncoordinated care.^14^ Achieving these outcomes may be facilitated by a comprehensive, holistic assessment of symptoms and their overall burden.^12,15,16^

Patient-reported outcome measures (PROMs), which are validated, self-reported measures of an individual's health, could support the monitoring of symptom burden in MLTC.^17,18^ Numerous symptom-focused PROMs (e.g. Edmonton Symptom Assessment Scale (ESAS)^19,20^, Asthma Control Questionnaire (ACQ)^21^ and Patient Health Questionnaire-9 (PHQ-9)^22^) have been developed for single conditions; however, symptom heterogeneity and MLTC complexity suggest these measures may not be content valid for MLTC. Moreover, the use of single-disease PROMs for MLTC is likely to require the administration of multiple PROMs to ensure symptom coverage within an individual. The need for multiple PROMs increases both administrative and respondent burden, and limits cross-comparison of data at the population or service level due to the necessity of tailoring PROMs to the combination of diseases present in each individual.^18^ Alternatively, generic symptom measures may not provide sufficient detail to support clinical management of MLTC.^23^ A PROM developed within a transdiagnostic paradigm to capture overall symptom burden independent of specific diagnoses could reduce treatment burden, promote efficiencies within services and enable holistic management by removing the need for multiple disease-specific metrics.^17,24^

The Symptom Burden Questionnaire™ for Long COVID (SBQ™-LC) is a modular, condition-specific PROM for assessment of symptom burden in people living with long COVID or post-COVID condition (PCC).^25^ It measures 123 symptoms grouped into 16 standalone scales organised by body system/function and a single seven-item scale measuring overall symptom interference (Impacts on Daily Life). The SBQ™-LC's organisation according to body system rather than by disease means it is well-positioned to be adapted for use as a transdiagnostic PROM of symptom burden in MLTC. In this study, we aimed to identify a set of shared concepts (symptoms) relevant for MLTC and develop a conceptual framework to adapt the SBQ™-LC for use as a novel, transdiagnostic PROM of MLTC symptom burden – the Symptom Burden Questionnaire™ for MLTC (SBQ™-MLTC).

## Methods

### Study design

This mixed-method, multi-phase concept elicitation study comprised: (1) concept identification, harmonisation and refinement, (2) evaluation of face validity by healthcare practitioners (HCPs) and (3) development of a conceptual framework.

### Phase 1: Concept elicitation, harmonisation and refinement

Concept elicitation is a foundational step in the development of PROMs. It involves systematically identifying concepts of interest, such as symptoms, disease impact and quality of life, that are most relevant to patients. Regulatory guidance recommends using qualitative methods to define the target construct based on first-hand accounts from individuals with lived experience.^26,27^ However, in MLTC, the use of traditional qualitative methods is challenging and resource-intensive due to the complexity and heterogeneity of the patient population.^28^ To address this challenge, we developed a list of 24 prevalent conditions in MLTC, drawing on the work of Fortin *et al.* (*n* = 20 conditions) and including four further conditions (i.e. hearing loss, visual impairment, alcohol misuse and eczema) due to their high prevalence^29,30^ (see Box 1). Reported MLTC prevalence varies widely, largely due to variation in the included conditions,^31^ and consistent definitions are needed to ensure comparable and consistent research in MLTC.^32^ Fortin *et al.*'s list was selected due to its reporting of higher and more stable estimates of MLTC prevalence, where the addition of further conditions had little impact.^30^ Relevant symptoms for each included condition were identified from two sources: (1) single disease-specific PROMs with evidence of patient involvement during concept elicitation and/or content validation and (2) a generative artificial intelligence (AI) large language model (LLM), ChatGPT 4.0.

Box 1. List of conditions (*n* = 24) prevalent in MTLCs used for concept elicitation based on Fortin *et al.*^29^

- Alcohol and substance misuse
- Arthritis
- Asthma, chronic obstructive pulmonary disease (COPD), emphysema or chronic bronchitis
- Cancer
- Cardiovascular disease (angina, myocardial infarction, atrial fibrillation, peripheral arterial disease)
- Chronic musculoskeletal conditions causing pain or functional limitation/back pain
- Chronic urinary problems (e.g. benign prostatic hyperplasia)
- Intestinal disorders (irritable bowel syndrome, inflammatory bowel disease, Crohn's disease, ulcerative colitis, diverticulosis, diverticular disease)
- Dementia or Alzheimer's disease
- Depression or anxiety
- Diabetes
- Eczema
- Gastric acid-related disorders (reflux, heartburn, gastric ulcer, gastritis)
- Hearing loss
- Heart failure
- Hyperlipidaemia (high cholesterol)
- Hypertension
- Kidney disease or failure
- Liver disease, chronic hepatitis
- Obesity
- Osteoporosis
- Stroke and transient ischaemic attack (TIA)
- Thyroid disorders
- Visual impairment, eye disorders

#### Concept elicitation from disease-specific PROMs

The ePROVIDE PROQOLID database is a comprehensive database of >8000 clinical outcome assessments (COAs) maintained by MAPI Research Trust (https://eprovide.mapi-trust.org/). PROQOLID was searched for relevant PROMs of symptom burden for the 24 conditions. Using the database's search filter functionality, searches were tailored for each condition by specifying ‘database’ AND ‘therapeutic indication’ AND ‘type of outcome assessment’, refined by ‘population’ and ‘language’ (see Supplementary file, Appendix 2 for the PROQOLID search strategy and eligibility criteria). Each PROM record in PROQOLID was reviewed. PROMs were included if they were developed with patient input through qualitative concept elicitation. The item content was reviewed, and constructs/symptoms were charted using a data extraction form in Microsoft Excel, with duplicates removed.

#### Concept elicitation through generative AI

ChatGPT is an LLM-based AI chatbot that uses a deep neural network architecture to generate human-like responses to specific prompts.^33^ For PROMs, generative AI has the potential to support concept elicitation by rapidly generating descriptions of target constructs. ChatGPT has been shown to be largely accurate in the generation of medical information; therefore, we were interested in examining its potential in developing the SBQ™-MLTC, where traditional qualitative concept elicitation studies would be resource-intensive due to the complexity and diversity of the patient population.^33,34^ Symptom lists (*n* = 24) were generated by ChatGPT 4.0 using prompts iteratively refined by the study team (MC, SH, BH) to maximise inclusivity and optimise the lists for sensitivity and specificity across the included conditions. ChatGPT was instructed to: ‘Give me a list of 75 most prevalent [*diabetes*] symptoms that are most impactful or burdensome to patients aged 18 years and over, of all ethnicities, races, genders and sexes from a range of socio-economic backgrounds without any duplicates’. Lists were exported to Excel for screening.

#### List harmonisation and refinement

Symptom lists from both data sources (i.e. single-disease PROMs and ChatGPT 4.0) were screened by three team members (BH, SH, NA). For each condition, duplicates, clinical signs and concepts unsuitable for patient-report were removed. PROQOLID and ChatGPT-generated lists were merged per condition and rescreened to remove duplicates. Next, symptoms were pooled across conditions and grouped by body system/function according to the conceptual framework of the source instrument, SBQ™-LC (see Appendix 3, Supplementary file). Each symptom was reviewed by the study team (MC, SEH (PRO methodologists) and SH, EH (general practitioners) against pre-specified eligibility criteria.^35^ List similarity was explored using descriptive statistics, reporting overlap (absolute and percentage) and calculating a Jaccard index for each condition. The Jaccard Index, as a measure of similarity between two datasets, ranges from 0 to 1, with higher values indicating greater similarity.

### Phase 2: Assessment of face validity by healthcare practitioners

To establish face validity, the draft symptom list and conceptual framework were circulated to healthcare practitioners (HCPs) from primary and secondary care. Specialities were purposively sampled to align with the framework's domains and recruited via known contacts and snowball sampling. HCPs were emailed the full list as a Microsoft Excel spreadsheet and invited to review domains relevant to their expertise. All HCPs reviewed the ‘Other symptoms’ and ‘Impacts on daily life’ domains. For each symptom, HCPs were asked to indicate whether it should be retained or excluded and to identify any additional MLTC-relevant symptoms missing from the list. Decisions were guided by the following criteria: (1) the SBQ™-MLTC should be broadly applicable to individuals living with MLTC; (2) included symptoms should have relevance for the clinical management of MLTC; and (3) considerations of inclusivity, including whether a symptom is of particular relevance to underserved groups and therefore should be included. Free-text fields allowed elaboration and additional insights.

HCPs’ responses were compiled for review by clinical team members (SH, EH, SEH). Descriptive statistics quantified consensus. Symptoms with unanimous endorsement from HCPs were automatically included. For symptoms lacking full HCP agreement, the team considered free-text comments to reach consensus. Items still without consensus were retained for patient evaluation during future cognitive debriefing. Qualitative analysis of the free text content was undertaken using the Framework method, applying a pre-specified deductive framework and generating further inductive codes.^36^

### Phase 3: Construction of the SBQ™-MLTC conceptual framework

Shortlisted symptoms were organised by body system or functional grouping to construct the SBQ™-MLTC's conceptual framework. Symptom assignment was clinically informed and based on domain relevance.

## Results

### Phase 1: Concept generation and refinement

#### Concept elicitation from disease-specific PROMs

Searches of PROQOLID, in July and August 2023, identified 338 PROMs with evidence of patient involvement in concept elicitation across the 24 health conditions. After removing duplicates (*n* = 159), 179 PROMs were screened, and 128 measures were removed after applying pre-specified eligibility criteria (see Supplementary file, Appendices 4 and 5). Fifty-one PROMs remained, yielding a total of 655 symptoms. The median number of PROMs per condition was five (range = 0 to 55). Cancer (*n* = 55) and diabetes (*n* = 27) were the conditions with the most PROMs. No PROMs were included for eight conditions (i.e. alcohol misuse, angina, Crohn's disease, hearing loss, hyperlipidaemia, hyperthyroidism, obesity and stroke).

#### Concept elicitation through generative AI

ChatGPT 4.0 generated 75 symptoms for each of the 24 conditions (*n* = 1800) between 20th July 2023 and 1st August 2023 (see Supplementary file, Appendix 6). After removing duplicates, clinical signs and symptoms unsuitable for patient report (*n* = 253), 1547 symptoms were retained.

#### List harmonisation and refinement

PROM-derived symptoms (*n* = 655) were merged with ChatGPT-generated symptom lists (*n* = 1547) to produce a list of 2202 symptoms for the 24 conditions. Jaccard indices ranged from 0 to 0.22 (mean = 0.06, SD = 0.07) as a measure of list similarity. Mean percentage of symptoms included in the PROMs symptom list and also present in ChatGPT-generated list was 21.68%, while 8.88% of symptoms generated by ChatGPT were covered by PROM-derived symptoms (see Supplementary file, Appendix 7). Following deduplication (*n* = 570), 1632 symptoms were sorted into 18 domains (i.e. 17 domains of SBQ™-LC's conceptual framework and an ‘unallocated symptoms’ domain).

**Figure 1.**
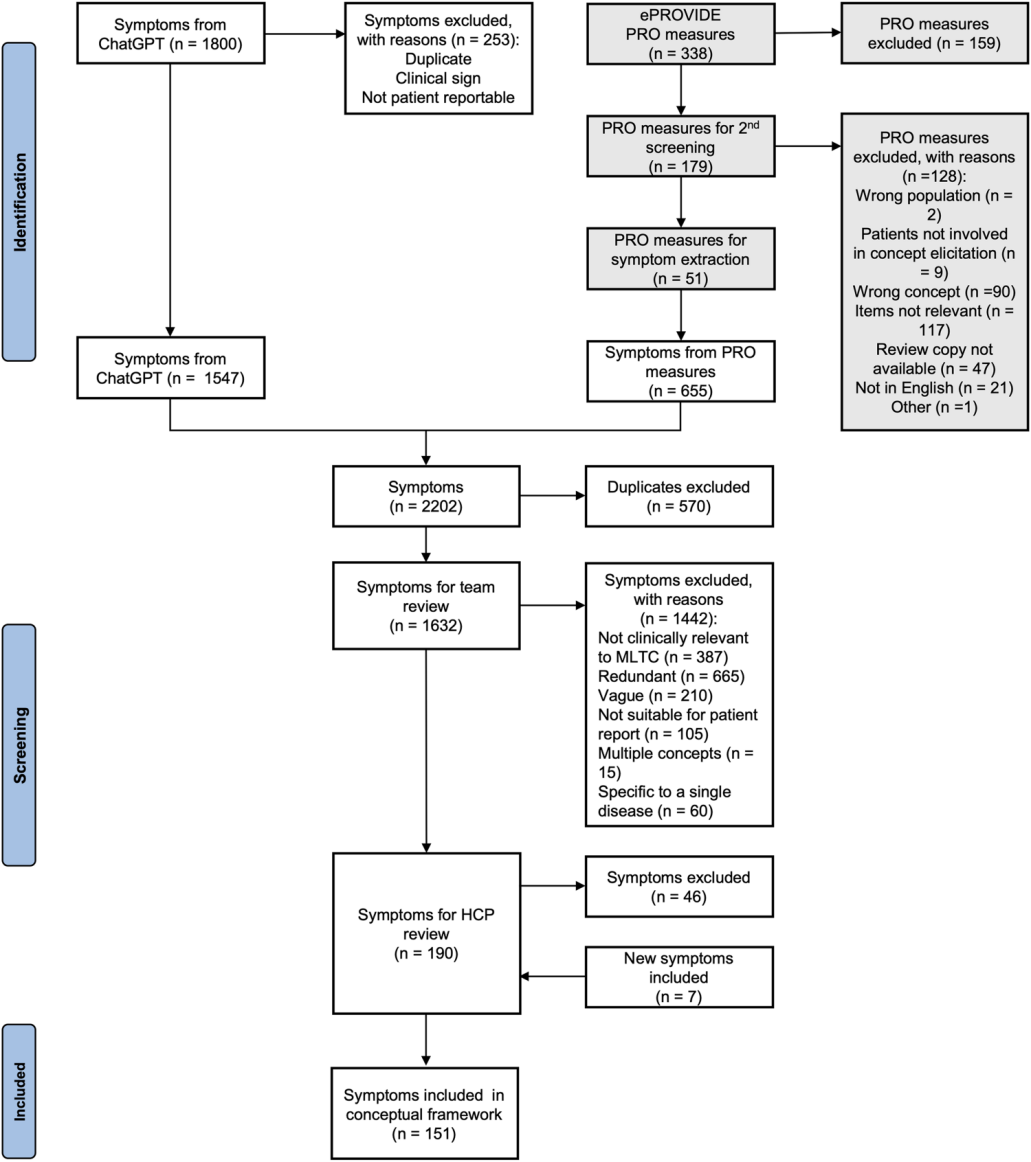
Flow diagram showing the process symptom identification, harmonisation and reduction.

Symptoms were reviewed by the study team (EH, MC, SH, SEH). Consensus removed 1442 symptoms, yielding 190 symptoms for HCP review. Symptoms were removed if already present in the list (i.e. redundant) (*n* = 665); not clinically relevant to MLTC (*n* = 387); vague (*n* = 210); unsuitable for patient-report (i.e. symptom/concept was a clinical sign or considered unsuitable for self-report) (*n* = 105); specific to a single disease (*n* = 60) or encompassed multiple constructs (*n* = 15) (see Supplementary file, Appendix 10). Figure 1 shows the flow diagram of symptom identification, harmonisation and reduction.

### Phase 2: Evaluation of face validity by healthcare practitioners

Twenty-eight HCPs were invited via email to review the draft symptom list (*n* = 190 items), and 17 HCPs participated between September 2024 and April 2025. Each domain was assessed by at least one HCP with relevant clinical expertise (see Supplementary file, Appendix 8), with 15/17 domains (88.2%) reviewed by two or more HCPs (median number of reviewers per symptom = 2, range 0–6). A minimum of two HCPs reviewed 174 of 190 symptoms (91.6%). Complete agreement (100%) was reached for 92 symptoms (52.9%). For symptoms without agreement (*n* = 82, 47.1%) and for any domain with only one HCP reviewer, clinical members of the study team (EH, SH), both general practitioners, examined the ratings and accompanying free-text comments to reach consensus on symptom eligibility through discussion. At the conclusion of Phase 2, 144 (74.7%) symptoms were retained, and seven new symptoms were added, yielding a final list of 151 symptoms (see Supplementary file, Appendix 9).

#### Qualitative analysis of free-text comments

A deductive framework was used to code free-text comments according to three primary themes: (1) symptom relevance to MLTC; (2) symptom comprehensiveness for the included conditions and (3) considerations for item development. HCPs noted when symptoms were relevant to MLTC (e.g. ‘Yes, [applies to] many rheumatological and systemic diseases’), when items overlapped with others in the list (e.g. ‘This overlaps with item 0199; I would recommend excluding or merging with 0199’). Symptoms of clinical concern were identified (e.g. ‘Red flag if duration exceeds four weeks’) and HCPs highlighted items associated with diminished quality of life or increased care needs (e.g. ‘A high metric of dependence’ and ‘Hearing impairment is socially isolating and associated with more rapid cognitive decline if no intervention’).

HCPs proposed additional symptoms to improve the comprehensiveness of the list and provided suggestions to support item development. For example, HCPs highlighted symptoms which used overly complex or confusing language (e.g. ‘… Most patients won't really tell you about this – I'm not sure how it will perform on a PRO’, and ‘it's more of a sign than a symptom and most people with dysfunctional breathing patterns don't realise they are hyperventilating – they'll describe it as [short of breath]’). Several HCPs provided suggestions relating to PROM design (e.g. ‘… In our domain, we would split fatigue and sleepiness as separate constructs. Given fatigue is covered in the next tab, I would recommend “daytime sleepiness” is one domain and “fatigue” is another’).

### Phase 3: Conceptual framework development

**Figure 2.**
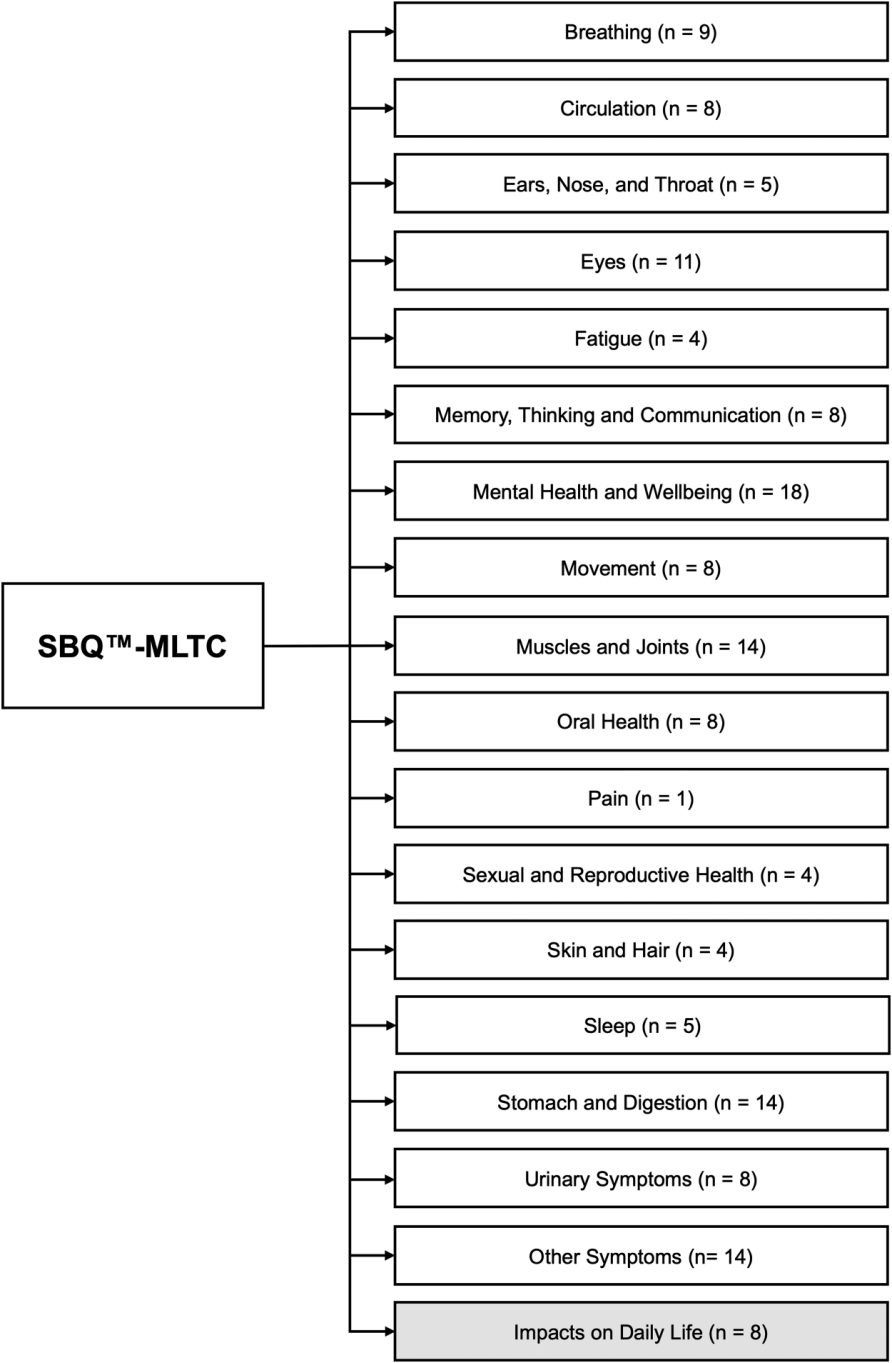
Conceptual framework of the SBQ™-MLTC.

A conceptual framework was constructed (see Figure 2), grouping the 151 shortlisted symptoms into 18 domains organised by body system or function, and including a cross-cutting domain capturing interference with daily activities (‘Impacts on Daily Life’). The framework included two additional domains (i.e. ‘urinary symptoms’ and ‘oral health’) not included in the source instrument (SBQ™-LC).

## Discussion

MLTC is associated with high symptom burden, manifesting as a variety of symptoms that affect multiple organ systems and impact multiple aspects of daily life. In this concept-elicitation study, we developed a conceptual framework for the SBQ™-MLTC, a new, transdiagnostic PROM of symptom burden applicable across 24 conditions highly prevalent in MLTC. The development of the SBQ™-MLTC directly addresses recommendations from a rapid evaluation of health service innovations for MLTC, which emphasised the importance of PROMs as a means of systematically capturing and monitoring the lived experiences of people with multiple long-term conditions.^18,23,37^

We also demonstrated the feasibility of a resource-efficient, mixed-method approach to concept elicitation. We triangulated data from a review of existing PROMs with symptom lists generated using a web-based generative AI tool. This novel approach, which also included content validation by HCPs, proved feasible and enabled efficient concept elicitation for a target construct (symptom burden) and population (people living with MLTC) characterised by complexity and heterogeneity.

### Strengths and limitations

The study employed a rigorous, mixed-method approach with data triangulation to ensure concept relevance and comprehensiveness; however, it also had limitations. Included conditions were based on a review of condition lists reported in the MLTC literature. Fortin *et al.*'s list was selected because it was derived through consensus and considered condition relevance to primary care services, impact on affected patients and prevalence in primary care.^29^ However, not all conditions prevalent in MLTC were included in the study (e.g. allergic rhinitis, migraine); therefore, the SBQ™-MLTC's conceptual framework may not fully capture symptom burden for some conditions. In such instances, we recommend further content validation studies to confirm whether the SBQ™-MLTC provides coverage of symptoms relevant to these patient groups. If coverage is incomplete, additional items should be generated.

It was not possible to validate the accuracy and authenticity of the symptom lists generated by ChatGPT4.0, as sources were not annotated in the model outputs. Algorithmic bias, arising from design, implementation or decision-making processes within the algorithms themselves, and the potential for bias arising from model inputs, can exacerbate existing social, cultural, or historical biases, leading to widening health disparities.^38^ Therefore, consistent with published literature on the use of AI in healthcare, we recommended generative AI tools not be used in isolation for PROM development and that all output undergo validation through triangulation with verifiable data sources, including clinical validation.^39^

We identified single-disease PROMs for the included conditions and extracted all symptoms, mapping these to LLM output. PROMs were identified from a single clinical outcome assessment (COA) database (i.e. PROQOLID) and searches of the academic literature were not undertaken. Therefore, relevant PROMs may not have been identified if these were not listed on PROQOLID. List triangulation and face validation by HCPs facilitated further refinement and item generation. Planned cognitive debriefing interviews with patients with MLTC will provide further opportunities to evaluate the SBQ™-MLTC's content validity.

### Implications for health care policy and practice

**Figure 3.**
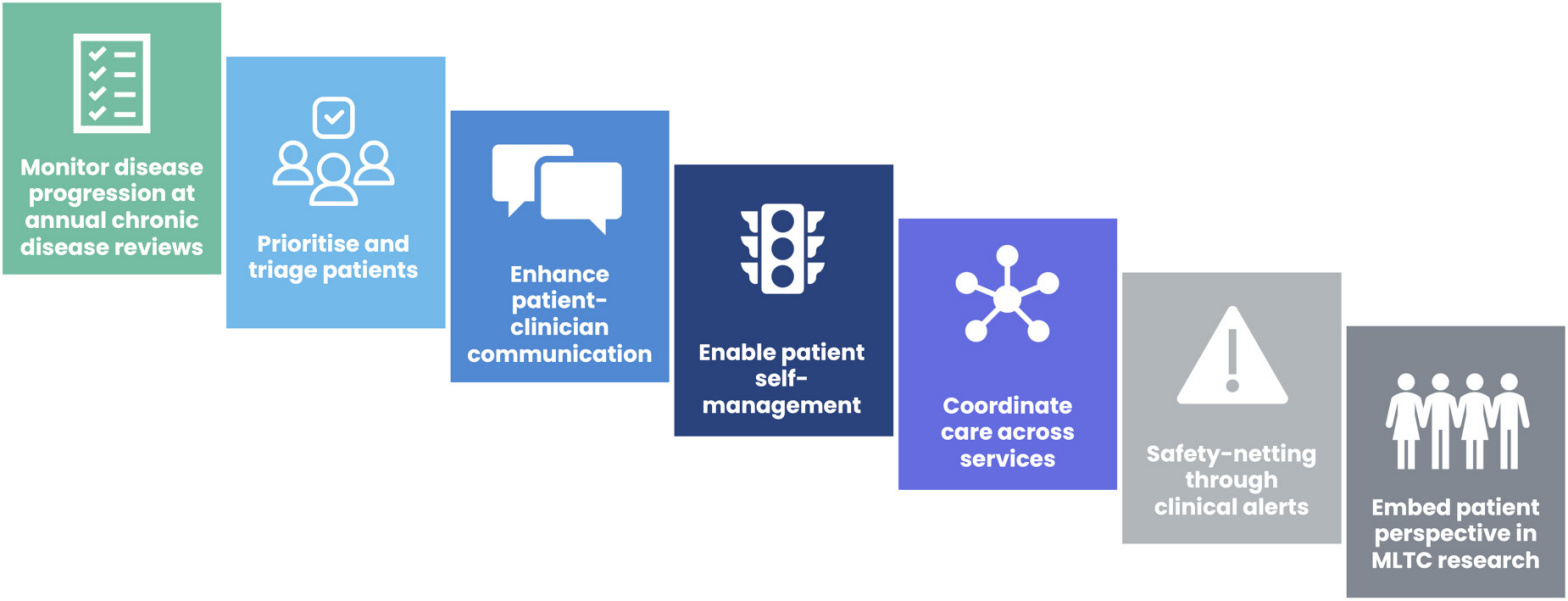
Value of PROs for MLTC care and research.

The self-reported nature of symptoms means that clinical management is often guided by ad hoc and unstructured accounts of patients’ experiences of symptoms. Despite this, patient-reported measurement of symptom burden in MLTC has received limited attention despite growing support for holistic, symptom-led rather than disease-led approaches to MLTC management.^9,12,37,40,41^ Symptom data collected using the SBQ™-MLTC could help identify and manage those symptoms that are most bothersome to patients (Figure 3). For example, completing SBQ™-MLTC in advance of primary care consultations and annual chronic disease reviews could support identification of disease progression and treatment side effects. If completed electronically with scores visible to both patients and clinicians (e.g. via patient-facing apps and integrated in the electronic health record), the SBQ™-MLTC could aid self-management, enhance communication with clinicians, provide safety-netting (e.g. alerts for adverse effects) and support shared decision-making and patient-centred care.^15,17,42^ Digital symptom monitoring using PROMs has been used successfully in secondary care to support triage of patients with single long-term conditions, reducing outpatient appointments.^43^ If deployed as a digital monitoring tool, the SBQ™-LC could offer a solution to target or rationalise GP workloads to where it is most needed rather than a one-size fits all approach.

MLTC is also associated with substantial treatment burden, often resulting from fragmented care and multiple treatment regimens. Individuals with MLTC and policymakers have emphasised the importance of holistic, patient-centred care.^14,44^ As a cross-cutting measure, the SBQ™-MLTC could support greater care integration by providing a common metric applicable across diseases, services and organisations, limiting duplication and improving communication.^45^

### Implications for MLTC research

In MLTC research, substantial effort has been directed toward identifying disease clusters, with the aim of advancing understanding of condition co-occurrence, shared mechanisms and pathophysiology and guiding intervention development.^46^ However, a disease-focused approach may underrepresent the lived experience of people with MLTC, where the cumulative impact of symptoms and drug interactions often contributes more directly to treatment burden and quality of life. A complementary perspective focuses on identifying symptom clusters that transcend diagnostic boundaries. The SBQ™-MLTC, constructed as a transdiagnostic PROM organised by body system and function, could support MLTC research involving large datasets. For example, SBQ™-MLTC scores could inform the development of prediction models and adverse event identification. At the service level, SBQ™-MLTC data could inform quality improvement initiatives and other service developments designed to optimise outcomes. SBQ™-MLTC data could also be used as a research tool to evaluate new interventions for MLTC (see Figure 3).

### Implications for PRO research

A growing body of literature is exploring the use of AI-based tools for PROM development. For example, studies have employed web scraping and machine-learning approaches to analyse patient online forums and social media, identifying PROMs that measure flare-ups in inflammatory bowel disease, supporting the development of a PROM for breast implant illness, and eliciting concepts relevant to measuring physical activity in chronic diseases.^47–49^ In this study, we explored the feasibility of using a web-based LLM (ChatGPT4.0) to elicit MLTC symptoms, which we triangulated with symptoms extracted from single-disease PROMs with evidence of patient involvement during their development. ChatGPT4.0 rapidly generated concepts; however, overlap between PROM items and the AI-generated lists was limited. This finding suggests a mixed-methods approach may be necessary when using generative AI, to ensure concept relevance and comprehensiveness for the target population. The findings also highlight the need for methodological research to identify key considerations for the use of generative AI for PROM development. Substantial dissimilarity across datasets also prompts consideration of whether qualitative approaches, the recommended ‘gold standard’, are sufficient. Whilst small, purposive qualitative samples enable in-depth exploration and rich descriptions, they are not designed to capture the diversity of patients, conditions or demographics required for representativeness. AI-based concept elicitation offers a complementary approach to capturing diversity and increasing representativeness and generalisability, thereby strengthening content validity with minimal additional resources.

## Conclusion

We identified relevant symptoms and constructed a conceptual framework for a novel, transdiagnostic PROM, the SBQ™-MLTC. Designed specifically to assess symptom burden, it is one of the few PROMs to be designed specifically for MLTC. Generative AI demonstrated potential for supporting PROM development for complex, heterogeneous patient populations. Future studies will include a qualitative study to construct and evaluate the SBQ™-MLTC through cognitive debriefing with patients and a cross-sectional survey study to evaluate its measurement properties. Once validated, the SBQ™-MLTC will be a patient-centred tool to support individualised patient management and advance research aimed at improving outcomes for people living with MLTCs.

## Supplemental Material

sj-pdf-1-shr-10.1177_20542704261459717 - Supplemental material for Symptom burden in multiple long-term conditions: An AI-supported, mixed-methods concept elicitation study Supplemental material, sj-pdf-1-shr-10.1177_20542704261459717 for Symptom burden in multiple long-term conditions: An AI-supported, mixed-methods concept elicitation study by Sarah E Hughes, Benjamin M A Hughes, Shamil Haroon, Eleanor Hathaway, Nicola Anderson, Christel McMullan, Michael Newnham, Clare Taylor, Alastair Denniston, Steven Backhouse, Andrew Filer, Karim Raza, Julia Scarisbrick, Clare Anderson, Matthew Lee, Lee Watts, Victoria Hodgetts Morton, Sarah Hillman, Faraz Mughal, Ed Day, Tariq Sami, Aliza Jesani, Thomas Jackson, Philip Collis, John Devin Peipert and Melanie Calvert in JRSM Open
