# Supplementary material for: Symptom burden in multiple long-term conditions: An AI-supported, mixed-methods concept elicitation study
Source: JRSM Open. 2026 Jul 15;17(7):20542704261459717. doi: 10.1177/20542704261459717 (PMC13373411; doi:10.1177/20542704261459717)
Supplement: sj-pdf-1-shr-10.1177_20542704261459717 - Supplemental material for Symptom burden in multiple long-term conditions: An AI-supported, mixed-methods concept elicitation study [file sj-pdf-1-shr-10.1177_20542704261459717.pdf]

Hughes, SE et al. Symptom burden in multiple long-term conditions: an AI-supported, mixed-methods concept elicitation study.

Supplementary File

Appendix 1: ePROVIDE PROQOLID[*] Search Strategy with exemplar screenshot 2

Appendix 3: PROMs identified through searching the ePROVIDE PROQOLID database for each of the 24 included conditions 4

Appendix 4: Screening of single-disease PROMs identified in ePROVIDE for each of the included 24 conditions 10

Appendix 5: Symptoms generated by ChatGPT-4.0 for the included conditions (N = 24) 11

Appendix 6: Jaccard indices as a measure of list similarity comparing symptoms extracted from condition-specific PROMs and ChatGPT 40

Appendix 7: Summary of pooled PROM and ChatGPT symptom list screening with reasons for symptom exclusion. Symptoms are organised by conceptual domains of the SBQ™-LC. 41

Appendix 8: Clinical specialities represented in the HCP sample (N = 17) 42

Appendix 9: Summary of HCP review and symptom shortlisting for each domain of the SBQ-LC (source instrument) conceptual framework 43

Appendix 10: Symptoms identified during concept elicitation 44

Appendix 1: ePROVIDE PROQOLID* Search Strategy with exemplar screenshot

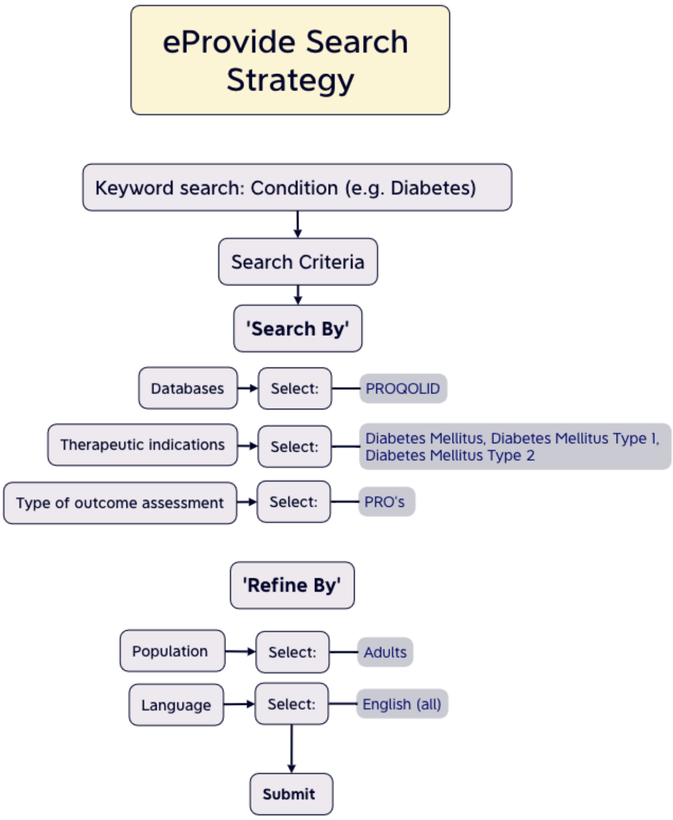

A. Flow diagram showing the ePROVIDE search strategy to locate disease-specific PROMs with diabetes as the exemplar condition.

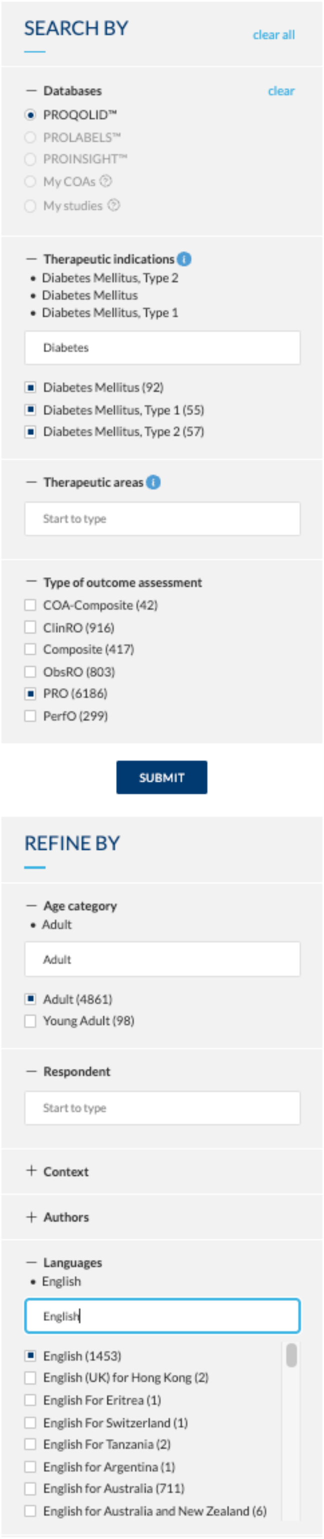

B. Illustrative screen shot showing the advanced search menu in ePROVIDE, applying the search strategy illustrated in A.

*https://eprovide.mapi-trust.org/advanced-search?database=proqolid

Appendix 2: Conceptual framework of the source instrument, the Symptom Burden Questionnaire™ for Long COVID (SBQ™-LC)

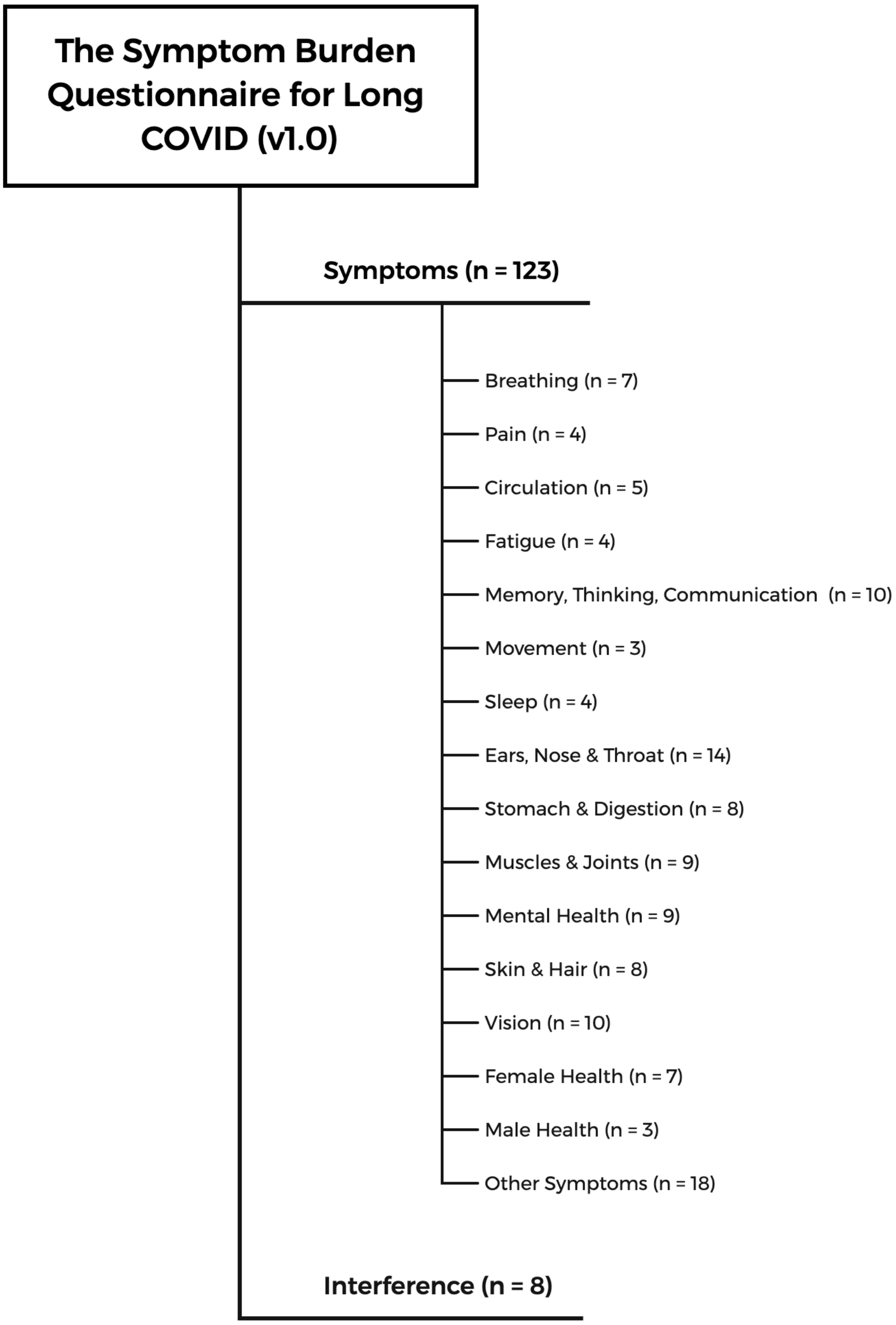

Reference: Hughes, SE, Haroon, S, McMullan, CM, Aiyegbusi, OL, Calvert, MJ. Symptom Burden Questionnaire™-Long COVID User Manual v1.1. ©2022 The University of Birmingham. All Rights Reserved.

Appendix 3: PROMs identified through searching the ePROVIDE PROQOLID database for each of the 24 included conditions

Condition	Name of PROM	Include/Exclude
Alcohol Misuse	No PROMs identified	N/A
Angina	No PROMs identified	N/A
Arthritis	RAID | Rheumatoid Arthritis Impact of Disease score	Exclude
	OHS | Oxford Hip Score	Exclude
	PsAQoL | Psoriatic Arthritis Quality of Life	Exclude
	BRAF-MDQ | Bristol Rheumatoid Arthritis Fatigue-Multidimensional Questionnaire	Exclude
	BRAF-NRS | Bristol Rheumatoid Arthritis Fatigue - Numerical Rating Scale	Exclude
	OKS | Oxford Knee Score	Exclude
	RA-WIS | Rheumatoid Arthritis Work Instability Scale	Exclude
	OSS | Oxford Shoulder Score	Exclude
	RAQoL | Rheumatoid Arthritis Quality of Life Questionnaire	Exclude
	OES | Oxford Elbow Score	Exclude
	FFI | Foot Function Index	Exclude
	RAOS | Rheumatoid and Arthritis Outcome Score	Include
	FLARE | Flare Assessment in Rheumatoid Arthritis	Exclude
Asthma	AQLQ | Asthma Quality of Life Questionnaire	Exclude
	SAC BDI-TDI | Self-administered Computerized version of the BDI-TDI	Exclude
	ALIS | Asthma Life Impact Scale	Exclude
	BHQ | Bronchial Hyperresponsiveness Questionnaire	Exclude
Cancer	BFI | Brief Fatigue Inventory	Exclude
	BREAST-Q™ | BREAST-Q™	Exclude
	CTSQ | Cancer Therapy Satisfaction Questionnaire	Exclude
	EORTC QLQ-BN20 | EORTC Quality of Life Questionnaire - Brain Cancer Module	Include
	EORTC QLQ-BR23 | EORTC Quality of Life Questionnaire - Breast Cancer Module	Include
	EORTC QLQ-COMU26 | EORTC Quality of Life Questionnaire - Communication	Exclude
	EORTC QLQ-CR29 | EORTC Quality of Life Questionnaire - Colorectal Cancer Module	Include
	EORTC QLQ-CX24 | EORTC Quality of Life Questionnaire - Cervical Cancer Module	Include
	EORTC QLQ-H&N35 | EORTC Quality of life - Head and Neck Cancer Module	Include
	EORTC QLQ-HCC18 | EORTC Quality of Life Questionnaire - Hepatocellular Carcinoma/Primary Liver Cancer Module	Include
	EORTC QLQ-INFO25 | EORTC Quality of Life Questionnaire - Information Module	Exclude
	EORTC QLQ-LC13 | EORTC Quality of Life Questionnaire - Lung Cancer Module	Include

Condition	Name of PROM	Include/Exclude
	EORTC QLQ-LMC21 | EORTC Quality of Life Questionnaire - Liver Metastases Colorectal Module	Exclude
	EORTC QLQ-NHL-HG29 | EORTC Quality of Life Questionnaire - Non Hodgkin Lymphoma High Grade Module	Include
	EORTC QLQ-OES18 | EORTC Quality of Life Questionnaire - Oesophageal Cancer Module	Include
	EORTC QLQ-OV28 | EORTC Quality of Life Questionnaire - Ovarian Cancer Module	Include
	EORTC QLQ-PAN26 | EORTC Quality of life Questionnaire - Pancreatic Cancer Module	Include
	EORTC QLQ-PR25 | EORTC Quality of Life Questionnaire - Prostate Cancer Module	Include
	EORTC QLQ-THY34 | EORTC Quality of Life Questionnaire - Thyroid Cancer	Include
	EORTC- QLQ-NHL-LG20 | EORTC Quality of Life Questionnaire - Non-Hodgkin Lymphoma Low Grade Module	Exclude
	EPIC-CP | Expanded Prostate Cancer Index Composite for Clinical Practice	Exclude
	FACIT-F | Functional Assessment of Chronic Illness Therapy - Fatigue	Exclude
	FACIT-Fatigue | Functional Assessment of Chronic Illness Therapy - Fatigue Scale	Exclude
	FACT-An | Functional Assessment of Cancer Therapy - Anemia	Exclude
	FACT-B | Functional Assessment of Cancer Therapy - Breast Cancer	Include
	FACT-Br | Functional Assessment Of Cancer Therapy - Brain	Include
	FACT-CNS | Functional Assessment of Cancer Therapy - Central Nervous System	Exclude
	FACT-Cog | Functional Assessment of Cancer Therapy - Cognitive function issues	Exclude
	FACT-ES | Functional Assessment of Cancer Therapy-Endocrine Subscale	Exclude
	FACT-G | Functional Assessment of Cancer Therapy - General	Include
	FACT-G Caregiver | Functional Assessment of Cancer Therapy - General - Caregiver	Exclude
	FACT-GOG-NTX12 | Functional Assessment of Cancer Therapy - Gynecologic Oncology Group-Neurotoxicity 12	Include
	FACT-ICM | Functional Assessment of Cancer Therapy – Immune Checkpoint Modulator	Include
	FACT-M | Functional Assessment of Cancer Therapy - Melanoma	Exclude
	FACT-MM | Functional Assessment of Cancer Therapy - Multiple Myeloma	Exclude
	FACT-P | Functional Assessment of Cancer Therapy - Prostate Cancer	Include
	FACT-VCI / FACT-Bl-Cys | Functional Assessment of Cancer Therapy - Vanderbilt Cystectomy Index / Functional Assessment of Bladder Cancer – Bladder Cystectomy	Include
	FKSI-15 | Functional Assessment of Cancer Therapy-Kidney Symptom Index	Include
	FLIC | Functional Living Index: Cancer	Exclude
	LCSS-Meso | Lung Cancer Symptom Scale-Mesothelioma	Exclude
	LYMPH-Q© | LYMPH-Q©	Exclude
	MAC | Mental Adjustment to Cancer Scale	Exclude
	MDASI-HF | MD Anderson Symptom Inventory - Heart Failure Module	Include
	MICRA | Multidimensional Impact of Cancer Risk Assessment	Exclude
	NEQ | Needs Evaluation Questionnaire	Exclude
	NFBrSI-24 | National Comprehensive Cancer Network-Functional Assessment of Cancer Therapy - Brain Symptom Index	Include

Condition	Name of PROM	Include/Exclude
	Oslo COVID-19 QLQ-W61© | Oslo COVID-19 health-related quality of life 61-item weekly questionnaire	Exclude
	QLI | Ferrans and Powers Quality of Life Index	Exclude
	QQ-q | Q(uality)-Q(uantity) questionnaire	Exclude
	QUOTE-GENECA | Quality of Care Through the Patient's Eyes - GENEtic counseling for hereditary CAncer	Exclude
	QWLQ-CS | Quality of Working Life Questionnaire for Cancer Survivors	Exclude
	RSCL | Rotterdam Symptom Checklist	Exclude
	SCFS-6 | Schwartz Cancer Fatigue Scale	Exclude
	SFSS | Structural-Functional Social Support Scale	Exclude
	UCLA-PCI | UCLA Prostate Cancer Index	Exclude
Crohn's Disease	CPWDQ | Crohn's disease Perceived Work Disability Questionnaire	Exclude
Dementia	D-QoL | Dementia Quality of Life Instrument	Exclude
	DEMQOL | Measurement of health-related quality of life for people with dementia	Include
	ZBI | Zarit Burden Interview	Include
	ZCI-AD-27 | Zarit Caregiver Interview for Alzheimer's Disease 27	Exclude
Depression	IDS-SR | Inventory of Depressive Symptomatology: Self-Report	Include
	PDQ-D | Perceived Deficits Questionnaire – Depression	Include
	SIQ | Sleep Inertia Questionnaire	Exclude
Diabetes	ADDQoL18 | Audit of Diabetes Dependent QoL 18	Exclude
	ADDQoL19 | Audit of Diabetes Dependent QoL 19	Include
	APPADL | Ability to Perform Physical Activities of Daily Living	Exclude
	CHES-Q | Current Health Satisfaction Questionnaire	Exclude
	DFS | Diabetic Foot Ulcer Scale	Exclude
	DiabMedSat | Diabetes Medication Satisfaction	Exclude
	DM-SAT | Diabetes Medication Satisfaction Questionnaire	Exclude
	DPM | Diabetes Productivity Measure	Exclude
	DQLCTQ | Diabetes Quality of Life Clinical Trial Questionnaire	Exclude
	DSAS-1 | Type 1 Diabetes Stigma Assessment Scale	Exclude
	DSAS-2 | Type 2 Diabetes Stigma Assessment Scale	Exclude
	DSC-R | Diabetes Symptom Checklist-Revised	Exclude
	DSM | Diabetes Symptom Measure	Include
	DSMQ | Diabetes Self-Management Questionnaire	Exclude
	DSQOLS | Diabetes specific quality of life scale	Exclude
	GME-Q | Glucose Monitoring Experiences Questionnaire	Exclude
	HypoA-Q | Hypoglycaemia Awareness Questionnaire	Exclude
	HypoA-Q Past month | Hypoglycaemia Awareness Questionnaire Past month	Exclude
	HypoA-Q SF | Hypoglycaemia Awareness Questionnaire Short Form	Exclude

Condition	Name of PROM	Include/Exclude
	ITEQ | Insulin Treatment Experience Questionnaire	Exclude
	ITSQ | Insulin Treatment Satisfaction Questionnaire	Exclude
	IW-SP | Impact of Weight on Self-Perception	Exclude
	IWQOL-Lite | Impact of Weight on Quality of Life - Lite	Exclude
	OHA-Q | Oral Hypoglycemic Agent Questionnaire	Exclude
	PAID | Problem Areas in Diabetes scale	Exclude
	QLI | Ferrans and Powers Quality of Life Index	Exclude
	SOADAS | Satisfaction with Oral Anti-Diabetic Agents Scale	Exclude
Eczema	PBI-HE | Patient Benefit Index - Chronic Hand Eczema	Exclude
Gastro-oesophageal Reflux Disease (GORD)	GERD-HRQL | Gastro-esophageal Reflux Disease Health Related Quality of Life scale	Exclude
	GIS | Gastro-oesophageal Reflux Disease (GERD) Impact Scale	Include
	GSAS | Gastroesophageal Reflux Disease Symptom Assessment Scale	Include
	NDI | Nepean Dyspepsia Index	Include
	PAGI-QoL | Patient Assessment of Upper Gastrointestinal Disorders-Quality of Life	Include
	PAGI-SYM | Patient Assessment of Upper Gastrointestinal Disorders Symptoms Questionnaire	Include
	QOLRAD | Quality Of Life in Reflux And Dyspepsia	Exclude
	RDQ | Reflux Disease Questionnaire	Exclude
	Reflux-Qual | Quality of Life Questionnaire in Gastroesophageal Reflux	Exclude
	RESQ-7 | Reflux Symptom Questionnaire, 7 day recall	Include
	RESQ-eD | Reflux Symptom Questionnaire e-Diary	Include
Hearing Loss	No PROMS identified	N/A
Heart Failure	CHQ / CHFQ | Chronic Heart Failure Questionnaire	Exclude
	KCCQ | Kansas City Cardiomyopathy Questionnaire	Exclude
	MacNew | MacNew Heart Disease Health-related Quality of Life Questionnaire	Exclude
	MDASI-HF | MD Anderson Symptom Inventory - Heart Failure Module	Include
	MLHF | Minnesota Living with Heart Failure Questionnaire	Exclude
Hyperlipidaemia	No PROMS identified	N/A
Hypertension	CAMPHOR | Cambridge Pulmonary Hypertension Outcome Review	Exclude
	emPHasis-10 | emPHasis-10	Include
	HTN BOS | Hypertension Battery of Scales (reduced)	Include
	LPH | Living with Pulmonary Hypertension Questionnaire	Include
	PAH-SYMPACT® | Pulmonary Arterial Hypertension-Symptoms and Impact (PAH-SYMPACT®) Questionnaire	Include
Hyperlipidaemia	No PROMS identified	N/A
Hyperthyroidism	No PROMS identified	N/A
Kidney Disease	CKD-AQ V2 | Chronic Kidney Disease-Anemia Questionnaire Version 2	Exclude
	ESRD-SCL-TM | End-Stage Renal Disease Symptom Checklist- Transplantation Module	Exclude

Condition	Name of PROM	Include/Exclude
	KDQOL-36™ Survey | Kidney Disease Quality of Life instrument™ - 36 items	Include
	KDQOL-SF™ | Kidney Disease Quality of Life instrument - Short form™	Include
	KDQOL™ | Kidney Disease Quality of Life instrument	Include
Liver Disease	CLDQ | Chronic Liver Disease Questionnaire	Include
	LDSI | Liver Disease Symptom Index	Exclude
Musculoskeletal	HAGOS | Copenhagen Hip and Groin Outcome Score	Include
	HAQ | Health Assessment Questionnaire	Exclude
	ÖMSQ | Örebro Musculoskeletal Screening Questionnaire	Exclude
	ÖMSQ-12 | Örebro Musculoskeletal Screening Questionnaire-12	Exclude
	OSIS | Oxford Shoulder Instability Score	Exclude
	PGQ | Pelvic Girdle Questionnaire	Exclude
	SMFA | Short Musculoskeletal Function Assessment	Include
	VISA-H | Victorian Institute of Sport Assessment-Proximal Hamstring Tendons questionnaire	Exclude
Obesity	APPADL | Ability to Perform Physical Activities of Daily Living	Exclude
	DAILY EATS | Daily Eats: Measuring Daily Eating Factors	Exclude
	IW-SP | Impact of Weight on Self-Perception	Exclude
	IWQOL-Lite | Impact of Weight on Quality of Life - Lite	Exclude
	IWQOL-Lite-CT© | Impact of Weight on Quality of Life-Lite Clinical Trials Version©	Exclude
	OWLQOL | Obesity and Weight-Loss Quality of Life measure	Exclude
	TRIM-W | Treatment Related Impact Measure - Weight	Exclude
	WRSM | Weight-Related Symptom Measure	Exclude
Osteoporosis	ADEOS | ADherence Evaluation of OSteoporosis treatment	Exclude
	OPTQoL | Osteoporosis-Targeted Quality of Life Questionnaire	Exclude
	QUALIOST® | QUAlity of Life questionnaire In OSTeoporosis	Include
Stroke	Neuro-QoL Item Bank v1.0 - Ability To Participate In Social Roles and Activities | Quality...	Exclude
	Neuro-QoL Item Bank v1.0 - Depression | Quality of Life in Neurological Disorders Item Ban...	Exclude
	Neuro-QoL Item Bank v1.0 - Fatigue | Quality of Life in Neurological Disorders Item Bank v...	Exclude
	Neuro-QoL Item Bank v1.0 - Lower Extremity Function - Mobility | Quality of Life in Neurol...	Exclude
	Neuro-QoL Item Bank v1.0 – Upper Extremity Function – Fine Motor, ADL	Exclude
	Neuro-QoL Short Form v1.0 - Ability to Participate in Social Roles and Activities | Qualit...	Exclude
	Neuro-QoL Short Form v1.0 - Fatigue | Quality of Life in Neurological Disorders Short Form...	Exclude
	Neuro-QoL Short Form v1.0 - Lower Extremity Function - Mobility | Quality of Life in Neuro...	Exclude
	QLI | Ferrans and Powers Quality of Life Index	Exclude
Urinary Incontinence	Contilife® | Quality of Life Assessment Questionnaire Concerning Urinary Incontinence	Exclude
	I-QOL | Urinary Incontinence-Specific Quality of Life	Exclude
	ICIQ- FLUTSsex | ICIQ-Female Sexual Matters associated with Lower Urinary Tract Symptoms	Exclude

Condition	Name of PROM	Include/Exclude
	ICIQ-FLUTS | International Consultation on Incontinence Questionnaire-Female Lower Urinary Tract Symptoms	Exclude
	ICIQ-FLUTS LF | International Consultation on Incontinence Questionnaire-Female Lower Urinary Tract Symptoms - Long Form	Exclude
	ICIQ-UI Short Form | International Consultation on Incontinence Questionnaire - Urinary Incontinence Short Form	Include
	IIQ | Incontinence Impact Questionnaire	Exclude
	IIQ-7 | Incontinence Impact Questionnaire - Short Form	Exclude
	SSI and SII | Symptom Severity Index and Symptom Impact Index for stress incontinence in women	Exclude
	USP | Urinary Symptom Profile	Exclude
Visual Impairment	MacTSQ | Macular Disease Treatment Satisfaction Questionnaire	Exclude
	NEI-VFQ-25 | National Eye Institute Visual Function Questionnaire-25	Include
	RetTSQs | Retinopathy Treatment Satisfaction Questionnaire (status)	Exclude
	VF-14 | Visual Function Index	Exclude
	VILL | Vision Impairment in Low Luminance Questionnaire	Exclude

Appendix 4: Screening of single-disease PROMs identified in ePROVIDE for each of the included 24 conditions

			Reason for exclusion							
**Condition**	**Number of unique PROMs identified**	**PROMs excluded**	**Wrong population**	**Patients not involved in concept elicitation**	**Wrong concept**	**Item stem not relevant**	**Review copy not available**	**Not in English**	**Other**	**PROMs included**
Alcohol Misuse	0	0	0	0	0	0	0	0	0	0
Angina	0	0	0	0	0	0	0	0	0	0
Arthritis	13	12	0	1	9	12	5	1	0	1
Asthma	4	4	0	0	4	4	2	1	0	0
Cancer	55	32	0	0	17	30	11	6	0	23
Crohn's Disease	1	1	1	1	0	0	0	0	0	0
Dementia	4	2	0	1	2	2	0	0	0	2
Depression	3	1	0	0	0	1	0	0	0	2
Diabetes	27	25	0	2	20	25	8	4	0	2
Eczema	1	1	1	0	1	1	1	1	0	0
GORD	11	4	0	0	2	4	1	1	1	7
Hearing Loss	0	0	0	0	0	0	0	0	0	0
Heart Failure	5	4	0	0	4	2	3	0	0	1
Hyperlipidaemia	0	0	0	0	0	0	0	0	0	0
Hypertension	5	1	0	0	0	1	0	0	0	4
Hyperthyroidism	0	0	0	0	0	0	0	0	0	0
Kidney Disease	5	2	0	1	1	2	1	1	0	3
Liver Disease	2	1	0	0	0	0	0	1	0	1
Musculoskeletal	8	6	0	0	5	3	2	1	0	2
Obesity	8	8	0	0	8	7	6	0	0	0
Osteoporosis	3	2	0	1	1	1	0	1	0	1
Stroke	9	9	0	0	5	9	1	0	0	0
Urinary Incontinence	10	9	0	1	8	9	5	2	0	**1**
Visual Impairment	5	4	0	1	3	4	1	1	0	**1**
**TOTAL**	**179**	**128**	2	9	90	117	47	21	1	**51**

Appendix 5: Symptoms generated by ChatGPT-4.0 for the included conditions (N = 24)

Condition: Arthritis

1. Adverse effects related to medication use
2. Altered biomechanics
3. Anaemia
4. Anxiety or depression due to chronic pain
5. Chest pain
6. Chronic cough
7. Chronic tiredness or fatigue
8. Cognitive difficulties, sometimes called "brain fog"
9. Crepitus (cracking or grating feeling or sound of bones)
10. Decreased life expectancy, primarily due to heart disease
11. Decreased muscle strength
12. Decreased range of motion in a joint or joints
13. Difficulty concentrating (sometimes related to chronic pain or fatigue)
14. Difficulty moving a joint or inability to move a joint
15. Difficulty sleeping due to pain
16. Difficulty swallowing
17. Difficulty urinating
18. Discoloured skin around affected joints
19. Disruption to social life
20. Dry mouth and eyes
21. Dry, itchy, or inflamed skin
22. Emotional distress
23. Eye pain, redness, or blurry vision
24. Fatigue
25. Fever
26. Fluid accumulation in the joints
27. Formation of bone spurs (extra bits of bone, which feel like hard lumps)
28. Frequent infections
29. Frequent urination
30. Gritty sensation in the eyes
31. Impaired growth in children and adolescents
32. Increase in cholesterol levels
33. Increase in fall risk due to joint instability
34. Increased risk of heart disease
35. Inflammation and damage to other body systems
36. Inflammation of the blood vessels
37. Joint damage that cannot be reversed
38. Joint deformity
39. Joint instability
40. Joint locking
41. Joint stiffness
42. Joint tenderness
43. Limping
44. Loss of appetite
45. Loss of coordination
46. Mild to severe discomfort when moving
47. Morning stiffness lasting for an hour or longer
48. Muscle weakness
49. Musculoskeletal pain
50. Nodules or lumps under the skin (rheumatoid nodules)
51. Numbness or tingling in the extremities
52. Osteoporosis
53. Pain in a joint or joints
54. Periods of increased disease activity (flares)
55. Problems with internal organs such as the heart or lungs
56. Problems with organ function due to systemic inflammation
57. Raynaud's phenomenon (spasms of small blood vessels in fingers and toes, changing colour)
58. Redness of the skin around a joint
59. Reduced aerobic fitness
60. Reduced flexibility
61. Reduced joint function
62. Reduced work productivity
63. Sensitivity to cold
64. Sensitivity to light
65. Sensitivity to weather changes
66. Sexual dysfunction
67. Shortness of breath

68. Sudden and unexplained skin rashes
69. Swelling in a joint or joints
70. Symmetrical symptoms (affecting both sides of the body equally)
71. Trouble with activities of daily living (ADLs)
72. Ulcers on the skin
73. Unexplained weight gain due to limited activity
74. Warmth of a joint
75. Weight loss

Condition: Angina (n = 75)

1. Abdominal pain
2. Anxiety or unease
3. Breathlessness upon exertion
4. Brittle or weak nails
5. Changes in skin colour (pale or blue coloration)
6. Changes in skin condition, such as discoloration or dryness
7. Changes in smell or loss of smell
8. Changes in taste or loss of taste
9. Changes in the shape of your toenails
10. Changes in vision
11. Chest pain or discomfort
12. Coldness in the legs or arms
13. Confusion or disorientation
14. Constipation
15. Darkened skin or skin discolorations
16. Decreased exercise tolerance
17. Decreased perception of temperature or pain
18. Decreased sexual drive
19. Difficulty concentrating or decreased alertness
20. Difficulty lifting objects or performing routine daily activities
21. Difficulty sleeping
22. Difficulty speaking or slurred speech
23. Difficulty standing after sitting for a long period
24. Difficulty swallowing
25. Dizziness
26. Dry or itchy skin
27. Enlarged neck veins
28. Erectile dysfunction in men
29. Fainting or near fainting
30. Fatigue
31. Feeling overly full soon after eating
32. Flushed or reddened skin
33. Fluttering or "thumping" feelings in the chest (heart palpitations)
34. Frequent urination
35. General sense of feeling unwell (malaise)
36. Hair loss on the legs and feet
37. Impaired balance or coordination
38. Increased sensitivity to cold or heat
39. Indigestion
40. Insomnia or difficulty staying asleep
41. Intolerance to cold temperatures, especially in the hands or feet
42. Intolerance to exercise
43. Irregular heartbeat
44. Light-headedness
45. Loss of appetite
46. Loss of consciousness
47. Memory problems or cognitive decline
48. Mood changes, including depression or anxiety
49. Muscle cramping or pain during physical activity
50. Nausea
51. Numbness or weakness in the legs or arms
52. Pain or discomfort during sexual intercourse
53. Pain or discomfort in the arms, neck, jaw, shoulder, or back
54. Persistent cough or wheezing
55. Pressure, tightness, or fullness in the chest
56. Rapid heart rate (tachycardia)
57. Rapid or irregular pulse
58. Restlessness, especially in the legs
59. Sensations of heat
60. Shortness of breath
61. Slow wound healing or infection
62. Slowed digestion
63. Snoring or sleep apnoea
64. Sores, ulcers, or infections on the legs or feet that won't heal
65. Sweating
66. Swelling in the legs, ankles, and feet (oedema)
67. Tenderness in the calf muscles
68. Thirst
69. Unexplained weight loss
70. Unusual or excessive nighttime urination
71. Visible blood vessels on the surface of the skin
72. Vomiting
73. Weak or absent pulse in the extremities
74. Weight gain due to fluid retention
75. Wheezing or shortness of breath during activity

Condition: Asthma (n = 75)

1. Abdominal pain or bloating
2. Altered taste sensation
3. Anxiety
4. Blue or grey lips or fingernails (cyanosis)
5. Changes in mood or emotions
6. Chest pain
7. Chest tightness
8. Chronic cough
9. Clubbing of the fingers
10. Cold, clammy skin
11. Confusion or forgetfulness
12. Decreased exercise tolerance
13. Decreased immune function
14. Decreased sexual activity
15. Depression
16. Difficulty climbing stairs
17. Difficulty performing daily tasks
18. Difficulty speaking in full sentences
19. Difficulty swallowing (dysphagia)
20. Difficulty taking a deep breath
21. Difficulty with physical exertion
22. Dry mouth
23. Excess mucus production
24. Eye problems, like dryness or redness
25. Fatigue
26. Flare-ups during changes in weather
27. Frequent awakening at night (nocturnal awakenings)
28. Frequent falls due to weakness or imbalance
29. Frequent hospitalizations or ER visits
30. Frequent respiratory infections
31. Frequent urination at night (nocturia)
32. Gastroesophageal reflux disease (GERD)
33. Hearing problems
34. Heart palpitations
35. Hoarseness or voice changes
36. Increased frequency of bone fractures
37. Increased heart rate (tachycardia)
38. Insomnia
39. Interrupted sleep
40. Intolerance to cold
41. Lack of appetite
42. Loss of bladder control
43. Loss of muscle mass
44. Low blood pressure (hypotension)
45. Low oxygen levels in the blood (hypoxemia)
46. Lower back pain
47. Malnutrition
48. Morning headaches
49. Morning stiffness and joint pain
50. Nail abnormalities
51. Neck vein distension
52. Night sweats
53. Noisy breathing (stridor)
54. Osteoporosis
55. Persistent cold symptoms
56. Poor growth and development in children
57. Poor work performance
58. Rapid breathing (tachypnoea)
59. Rapid heartbeat or palpitations
60. Reduced quality of life
61. Restlessness
62. Sensitivity to allergens or irritants (smoke, pollen, pet dander, etc.)
63. Sensitivity to cold or heat
64. Shortness of breath (dyspnoea)
65. Sinusitis
66. Skin discoloration or bruising
67. Social isolation due to fear of breathlessness
68. Swelling of the legs or feet (peripheral oedema)
69. Swollen ankles, feet, or legs (oedema)
70. Throat irritation
71. Trouble concentrating
72. Trouble sleeping due to breathing difficulties
73. Unintentional weight gain
74. Weight loss
75. Wheezing

Condition: Alcohol/substance misuse (n = 75)

1. Anxiety attacks.
2. Anxiety.
3. Blackouts or memory loss.
4. Bruises or infections at injection sites (for intravenous drug users).
5. Changes in academic or work performance.
6. Changes in appetite, either increased or decreased.
7. Changes in personal grooming habits.
8. Changes in personality.
9. Chest pain.
10. Chronic fatigue or lethargy.
11. Constant restlessness.
12. Continual use despite negative consequences.
13. Decreased sex drive.
14. Dental problems (e.g., tooth decay from methamphetamine use).
15. Depression.
16. Difficulty concentrating or remembering.
17. Difficulty in maintaining personal relationships.
18. Engagement in illegal activities to obtain the substance.
19. Engaging in risky behaviours (like driving under influence).
20. Feelings of worthlessness or excessive guilt.
21. Financial instability.
22. Flushing of the skin.
23. Frequent legal issues.
24. Frequent nosebleeds (possible sign of snorted drugs).
25. Hallucinations or delusions.
26. High blood pressure.
27. Impaired coordination or motor functions.
28. Impaired immune system.
29. Inability to stop using despite wanting to.
30. Increased risk of accidents or injuries.
31. Increased tolerance (needing more to feel the same effects).
32. Intense cravings for the substance.
33. Irregular heart rate.
34. Irritability or agitation.
35. Isolation or withdrawal from social activities.
36. Kidney disease.
37. Lack of interest in hobbies or activities once enjoyed.
38. Legal troubles related to substance use.
39. Liver disease.
40. Mental confusion.
41. Mood swings.
42. Nausea and vomiting.
43. Neglected appearance.
44. Neglecting personal hygiene.
45. Neglecting responsibilities at work, school, or home.
46. Neglecting to eat or overeating.
47. Overdose.
48. Panic attacks.
49. Paranoia or excessive fear without reason.
50. Periods of hyperactivity or manic behaviour.
51. Persistent cough or frequent bronchitis (in case of smoking substances).
52. Persistent headaches.
53. Persistent runny nose or sniffling.
54. Physical dependence on the substance.
55. Psychosis (loss of touch with reality).
56. Rapid or dramatic weight changes.
57. Rapid or rambling speech.
58. Red, bloodshot, or glassy eyes.
59. Relationship problems (fights, breakups).
60. Sexual dysfunction.
61. Skin changes (pale, flushed, or jaundiced skin).
62. Sleep disturbances (insomnia, oversleeping).
63. Slurred speech.
64. Stomach and digestion problems.
65. Sudden changes in friend groups or hangout places.
66. Suicidal thoughts or actions.
67. Sweating.
68. Tremors or seizures without a pre-existing condition.
69. Uncharacteristic dishonesty or secrecy.
70. Unexplained financial problems.
71. Unexplained injuries or accidents.
72. Unusual hyperactivity or agitation.
73. Unusual odours on breath, body, or clothing.
74. Weight loss or gain unrelated to other health conditions.
75. Withdrawal symptoms when not using the substance.

Condition: Cancer (n = 75)

1. Abdominal pain or discomfort
2. Balance problems
3. Bloating
4. Blood in the stool
5. Bone pain or fractures
6. Change in bowel habits
7. Change in sense of smell
8. Changes in mole appearance or new skin growths
9. Changes in the mouth or tongue
10. Changes in the shape, size, or colour of a mole or freckle
11. Chest pain
12. Cold hands or feet
13. Confusion or other mental changes
14. Constipation
15. Diarrhoea
16. Difficulty breathing
17. Difficulty concentrating
18. Difficulty urinating or changes in urine
19. Difficulty walking
20. Dizziness or fainting
21. Easy bruising or bleeding
22. Edema (swelling in legs or ankles)
23. Enlarged liver or spleen
24. Excessive sweating or flushing
25. Fatigue
26. Feeling full quickly after eating
27. Fever
28. Flushed or reddened skin
29. Frequent infections
30. Frequent or persistent infections
31. General weakness or lethargy
32. Gum bleeding
33. Headaches
34. Hearing loss or changes
35. High blood pressure or changes in heart rate
36. Hoarseness
37. Increased thirst or urination
38. Insomnia or other sleep disturbances
39. Jaundice (yellowing of the skin and eyes)
40. Loss of appetite or changes in taste
41. Memory problems or changes in cognitive function
42. Menstrual changes
43. Mood changes, such as depression or anxiety
44. Muscle pain or weakness
45. Nausea
46. Night sweats
47. Nosebleeds
48. Pain or discomfort during sex
49. Pain that does not go away
50. Paleness or pallor
51. Persistent cough
52. Persistent itchiness without rash
53. Petechiae (small red spots under the skin)
54. Red or purple skin patches
55. Seizures
56. Sexual dysfunction
57. Shortness of breath
58. Skin changes (colour, itchiness, redness)
59. Skin ulcers
60. Slow healing of wounds
61. Sores that don't heal
62. Swelling or fluid build-up
63. Swollen lymph nodes
64. Swollen or painful joints
65. Thickened, pitted, or ridged nails
66. Tingling or numbness in extremities
67. Trouble controlling bowel or bladder function
68. Trouble swallowing
69. Unexplained fractures
70. Unexplained muscle or joint pain
71. Unexplained weight loss
72. Unusual bleeding or bruising
73. Unusual hair growth or loss
74. Vision changes
75. Vomiting

Condition: Crohn's Disease (n = 75)

1. Abdominal pain
2. Abscesses
3. Anal fissures
4. Anaemia (due to chronic blood loss in stool)
5. Anxiety
6. Aphthous stomatitis (mouth ulcers)
7. Arthritis (joint pain)
8. Back pain
9. Bloating
10. Bloody stools
11. Bruising or bleeding easily
12. Changes in skin colour
13. Changes in skin texture
14. Changes in taste or smell
15. Changes in vision
16. Changes in weight (gain or loss)
17. Constipation
18. Dehydration
19. Delayed puberty (in adolescents)
20. Delayed wound healing
21. Depression
22. Diarrhoea
23. Difficulty concentrating (cognitive dysfunction)
24. Difficulty sleeping (insomnia)
25. Difficulty swallowing
26. Difficulty with coordination or balance
27. Dizziness
28. Dry eyes or mouth
29. Dyspepsia (indigestion)
30. Erectile dysfunction
31. Excessive sweating
32. Eye inflammation (uveitis, episcleritis)
33. Fatigue
34. Feeling cold all the time
35. Fever
36. Fistulas (abnormal connections between different body parts)
37. Flatulence (excessive gas)
38. Frequent urination
39. Gallstones
40. Growth failure (in adolescents)
41. Hair loss
42. Haemorrhoids
43. Incomplete evacuation (feeling of still needing to go after a bowel movement)
44. Increased susceptibility to infections
45. Infertility
46. Irregular heartbeat
47. Irregular menstrual cycle
48. Kidney stones
49. Liver disease (Primary Sclerosing Cholangitis)
50. Loss of appetite
51. Malnutrition (due to malabsorption)
52. Memory problems
53. Mucus in stool
54. Muscle weakness
55. Nausea
56. Night blindness
57. Night sweats
58. Oral ulcers (canker sores)
59. Osteoporosis (weakened bones)
60. Peripheral neuropathy (numbness or tingling in extremities)
61. Rectal bleeding
62. Reduced libido (sexual drive)
63. Reduced physical endurance
64. Sensitivity to noise or light
65. Severe headaches
66. Shortness of breath
67. Skin problems, such as erythema nodosum
68. Stomach cramps
69. Swollen gums
70. Swollen lymph nodes
71. Tinnitus (ringing in the ears)
72. Unintended weight loss
73. Unusually fast or slow heart rate
74. Urgency to defecate
75. Vomiting

Condition: Dementia (n = 75)

1. Agitation
2. Anxiety
3. Apathy
4. Becoming easily overwhelmed
5. Changes in appetite
6. Changes in mood and personality
7. Changes in sensory perception
8. Confusion about time or place
9. Decline in ability to complete normal tasks
10. Decreased motivation
11. Decreased or poor judgment
12. Delusions or false beliefs
13. Depression
14. Difficulty adapting to change
15. Difficulty concentrating
16. Difficulty dressing appropriately
17. Difficulty finding the right words
18. Difficulty following conversations
19. Difficulty handling money and bills
20. Difficulty identifying faces
21. Difficulty interpreting cues from the environment
22. Difficulty managing stress
23. Difficulty recognizing common objects
24. Difficulty recognizing family and friends
25. Difficulty swallowing
26. Difficulty with coordination and motor functions
27. Difficulty with planning and organization
28. Difficulty with problem-solving
29. Difficulty writing or reading
30. Disorientation in familiar places
31. Emotional flatness (reduced expressiveness)
32. Excessive adherence to routines
33. Forgetting the meaning of numbers
34. Getting lost in familiar places
35. Hallucinations
36. Hoarding behaviour
37. Impulsive behaviour
38. Inability to carry out normal daily tasks
39. Inability to follow directions
40. Inability to learn new things
41. Inability to perceive temperature
42. Inability to sense when they are hungry or full
43. Incontinence (loss of bladder or bowel control)
44. Increased susceptibility to infections
45. Indifference to other people's emotions
46. Insomnia or sleep disturbances
47. Lack of personal hygiene
48. Lack of spatial awareness
49. Loss of empathy
50. Loss of initiative and spontaneity
51. Loss of mathematical skills
52. Loss of reasoning skills
53. Loss of taste or smell
54. Memory loss
55. Misinterpreting sensory information
56. Misplacing items frequently
57. Neglecting hobbies and interests
58. Neglecting personal safety
59. Paranoia
60. Physical and verbal aggression
61. Poor insight into their condition
62. Problems with balance and falls
63. Reduced attention span
64. Reduced pain perception
65. Repeating questions or phrases
66. Repetitive behaviour or speech
67. Resistance to caregiving
68. Restlessness
69. Socially inappropriate behaviour
70. Sundowning (increased confusion and agitation in the evening)
71. Trouble understanding visual images and spatial relationships
72. Unexplained weight loss or gain
73. Unusual emotional reactions
74. Wandering and getting lost
75. Withdrawal from social activities

Condition: Depression (n = 75)

1. A change in personal performance at work, school, or social engagements
2. A need for a very rigid daily routine
3. A pattern of avoiding social interactions
4. Appetite and/or weight changes
5. Avoiding situations due to perceived danger or fear
6. Avoiding situations that make you feel anxious
7. Being easily fatigued
8. Chronic indigestion
9. Cold, sweaty, numb, or tingling hands or feet
10. Compulsive behaviours
11. Constantly feeling upset or agitated
12. Decreased energy or fatigue
13. Desire for isolation
14. Desire to control everything
15. Difficulty concentrating, remembering, making decisions
16. Difficulty controlling worry
17. Difficulty handling uncertainty
18. Disproportionate fear of specific things or situations
19. Dizziness
20. Dry mouth
21. Excessive concern about future events
22. Excessive fear of being judged or scrutinized by others
23. Excessive worry about various topics, events, or activities
24. Fear of losing control
25. Fear of losing your mind
26. Fear of making mistakes
27. Fear of social situations
28. Feeling a loss of control
29. Feeling anxious to the point of passivity
30. Feeling constantly overwhelmed
31. Feeling hopeless or pessimistic
32. Feeling of impending doom
33. Feeling overwhelmed
34. Feeling weak or tired
35. Feelings of detachment
36. Feelings of guilt, worthlessness, or helplessness
37. Flashbacks to traumatic events
38. Frequent crying for no apparent reason
39. Gastrointestinal problems
40. Heart palpitations
41. Hypervigilance
42. Hypochondria
43. Inability to be still and calm
44. Inability to enjoy quiet time or relax
45. Inability to experience joy
46. Inability to focus or concentrate
47. Inability to let go of worry
48. Insomnia, early-morning awakening, or oversleeping
49. Intrusive thoughts about a traumatic event
50. Irrational fear
51. Irritability
52. Lack of motivation
53. Loss of interest or pleasure in hobbies and activities
54. Muscle tension
55. Nausea
56. Nervousness
57. Obsessive thoughts
58. Overcompensating or overdoing things
59. Overthinking
60. Overwhelming feelings of uncertainty
61. Overwhelming need for reassurance
62. Panic attacks
63. Perfectionism
64. Persistent nightmares
65. Persistent sad or "empty" mood
66. Persistent self-doubt
67. Physical ailments without a clear cause (like aches, pains)
68. Procrastination due to fear of failure
69. Repeatedly going over thoughts
70. Restlessness, feeling wound-up, or on edge
71. Self-doubt
72. Shortness of breath
73. Tense muscles
74. Thoughts of death or suicide; suicide attempts
75. Trouble sleeping

Condition: Diabetes (n = 75)

1. Altered mental status
2. Blurred vision
3. Breath that smells fruity (due to ketoacidosis)
4. Changes in menstruation
5. Changes in skin colour
6. Changes in the vision, like floaters
7. Chest pain
8. Chronic constipation or diarrhoea
9. Chronic skin conditions, like bacterial or fungal infections
10. Cognitive decline
11. Compromised immunity
12. Darkened skin in areas of body creases (Acanthosis nigricans)
13. Dehydration
14. Dental problems (Periodontitis)
15. Depression or anxiety
16. Difficulty concentrating
17. Difficulty swallowing
18. Difficulty understanding speech
19. Dry mouth
20. Erectile dysfunction
21. Excessive thirst (Polydipsia)
22. Fatigue or tiredness
23. Foot problems, like ulcers or gangrene
24. Frequent colds or flu
25. Frequent headaches
26. Frequent infections
27. Frequent urination (Polyuria)
28. Frequent urination at night (Nocturia)
29. Fruity-smelling breath
30. Gastroparesis (Delayed stomach emptying)
31. Hair loss on the lower extremities
32. Hand syndrome (Limited joint mobility)
33. Hearing impairment
34. Heat intolerance
35. High blood pressure
36. High cholesterol
37. Increased bruising
38. Increased heart rate (Tachycardia)
39. Increased susceptibility to diseases
40. Intense hunger (Polyphagia)
41. Irregular heartbeat (Arrhythmia)
42. Irritability
43. Itchy skin
44. Joint pain or stiffness
45. Loss of bladder control
46. Loss of coordination and balance
47. Mood swings
48. Nausea and vomiting
49. Night sweats
50. Non-alcoholic fatty liver disease
51. Non-healing wounds on feet
52. Numbness or tingling in the hands or feet (Peripheral neuropathy)
53. Osteoporosis
54. Pain or discomfort in the abdomen
55. Persistent bad breath
56. Persistent hunger even after eating
57. Poor circulation
58. Poorly controlled blood glucose
59. Rapid breathing (Hyperventilation)
60. Recurrent yeast infections
61. Reduced sexual desire
62. Restless leg syndrome
63. Seizures
64. Shaking or trembling
65. Shortness of breath
66. Skin tags
67. Sleep apnoea
68. Slow healing after dental procedures
69. Slow-healing sores or cuts
70. Swollen ankles, feet, and legs (Edema)
71. Unconsciousness or coma (in severe cases)
72. Unexplained muscle loss
73. Unexplained weight loss
74. Unusual drowsiness or lethargy
75. Unusual weight gain

Condition: Eczema (n = 75)

1. Allergic reactions to certain substances
2. Avoidance of social activities due to visible symptoms
3. Avoidance of swimming or other water activities due to chlorine sensitivity
4. Changes in skin pigmentation
5. Decreased productivity at work or school due to discomfort
6. Depressive symptoms related to chronic discomfort
7. Development of callouses on frequently scratched areas
8. Development of hand eczema due to frequent washing
9. Development or worsening of asthma or hay fever
10. Diet restrictions due to food sensitivities
11. Difficulty concentrating due to discomfort or itchiness
12. Difficulty finding effective treatments
13. Difficulty in balancing the impact of the condition with daily life responsibilities.
14. Difficulty in maintaining a skincare routine during travel
15. Difficulty managing the condition alongside other health issues
16. Difficulty sleeping due to itchiness
17. Difficulty with intimate relationships due to skin sensitivity or self-consciousness
18. Discoloured skin patches
19. Dry skin
20. Exacerbation of symptoms due to hormonal changes (like during menstruation)
21. Exacerbation of symptoms with alcohol consumption
22. Feelings of hopelessness due to chronic nature of the condition
23. Feelings of self-consciousness or embarrassment
24. Financial burden due to treatment costs
25. Flare-ups triggered by stress or emotional upset
26. Frequent skin checks or medical visits
27. Frequent skin infections
28. Impact on mental health and overall wellbeing
29. Inability to partake in certain physical activities due to skin irritation
30. Increased bathing or showering frequency to manage symptoms
31. Increased health insurance costs due to regular doctor visits
32. Increased laundry frequency due to skin irritation from worn clothing
33. Increased sensitivity to environmental allergens
34. Increased susceptibility to other skin conditions
35. Infected skin sores
36. Inflammation
37. Itchy skin
38. Limited clothing choices due to skin sensitivity
39. Limited occupational choices due to skin sensitivities
40. Lowered quality of life due to ongoing discomfort
41. Lowered self-esteem due to physical appearance
42. Mood changes such as irritability or frustration
43. Necessity to change lifestyle habits to manage condition
44. Need for constant skincare management
45. Need for frequent moisturizing
46. Need for special bedding or materials due to skin irritation
47. Oozing or weeping lesions
48. Overheating due to needing to wear long sleeves or pants to hide skin
49. Painful skin
50. Psychological distress
51. Red or brownish-grey patches
52. Restlessness
53. Risk of eye problems like conjunctivitis or eyelid dermatitis
54. Risk of skin scarring
55. Rough or leathery skin texture
56. Sensitivity to certain materials or fabrics
57. Sensitivity to certain personal care products, like soaps or shampoos
58. Sensitivity to cold or dry weather
59. Sensitivity to environmental triggers
60. Sensitivity to heat or sweating
61. Sensitivity to specific foods
62. Skin thickening over time due to scratching
63. Small, raised bumps that leak fluid when scratched
64. Social isolation due to feelings of difference or embarrassment
65. Stigmatization or bullying due to visible symptoms
66. Stinging or burning sensation when applying certain creams or lotions
67. Stress or anxiety due to the condition
68. Struggle with body image due to visible symptoms

69. Struggles with temperature regulation due to sensitivity to heat and sweat
70. Swelling
71. Thickened, cracked, or scaly skin
72. Time-consuming daily skincare routine
73. Tiredness or fatigue due to sleep disruption
74. Workplace or school absences due to severe flare-ups
75. Worsening of symptoms with age

Condition: Gastro-Oesophageal Reflux Disease (GORD) (n = 75)

1. Abdominal pain or discomfort
2. Acid reflux
3. Altered taste sensation
4. Anaemia (due to blood loss)
5. Anxiety about eating due to fear of pain
6. Asthma symptoms (if the stomach acid backs up into the lungs)
7. Bad breath (halitosis)
8. Bitter taste in the mouth
9. Black or bloody stools
10. Bloating
11. Changes in bowel habits (constipation or diarrhoea)
12. Chest pain
13. Chronic throat clearing
14. Cold hands and feet (related to anaemia)
15. Constant hunger
16. Dark, tarry stools (melena, a sign of gastrointestinal bleeding)
17. Decreased interest in food
18. Decreased muscle strength (related to malnutrition or dehydration)
19. Decreased productivity at work due to symptoms
20. Decreased tolerance for exercise
21. Dehydration (from vomiting or decreased fluid intake)
22. Dental erosions or cavities (due to the acid)
23. Depression due to chronic symptoms
24. Difficulty concentrating due to symptoms
25. Difficulty sleeping due to symptoms (insomnia)
26. Difficulty swallowing (dysphagia)
27. Disrupted social activities due to symptoms
28. Dizziness or light headedness (related to dehydration or anaemia)
29. Earache
30. Excessive burping or belching
31. Fatigue
32. Feeling full quickly when eating
33. Feeling of a lump in the throat
34. Feeling of excessive fullness after meals
35. Gagging or choking
36. Gas
37. Gastrointestinal bleeding
38. Heartburn
39. Hoarseness or laryngitis
40. Indigestion
41. Involuntary weight loss
42. Irregular heartbeat
43. Jaundice (yellowing of the skin or eyes, a sign of severe disease)
44. Loss of appetite
45. Nausea
46. Nervousness or anxiety due to symptoms
47. Night sweats (from discomfort or pain)
48. Pain or discomfort in the upper abdomen
49. Pain that worsens with empty stomach
50. Pale stools
51. Palpitations
52. Persistent dry cough
53. Poor quality of life due to symptoms
54. Postnasal drip
55. Reduced enjoyment of food due to symptoms
56. Reduced physical activity due to symptoms
57. Regurgitation (a sensation of acid backing up into the throat)
58. Salivation (increased saliva production)
59. Sensation of food stuck in the throat
60. Shortness of breath
61. Sinusitis
62. Skin pallor (related to anaemia)
63. Sore throat
64. Sour taste in the mouth
65. Stomach noises (rumbling, gurgling, growling)
66. Ulcer pain
67. Unintentional weight loss
68. Upset stomach (dyspepsia)
69. Vomiting
70. Vomiting blood (hematemesis, a sign of gastrointestinal bleeding)
71. Waking up at night due to symptoms (nocturnal awakening)
72. Water brash (a rush of saliva after an episode of regurgitation to dilute the acid in the oesophagus)
73. Wheezing
74. Worsening of symptoms after meals
75. Worsening of symptoms when lying down

Condition: Hearing loss (n = 75)

1. Anxiety in social situations due to hearing impairment.
2. Avoidance of some social settings.
3. Challenges in maintaining independence.
4. Decreased job performance due to communication difficulties.
5. Depression or feelings of isolation.
6. Difficulty enjoying music or other auditory arts.
7. Difficulty following along with audiobooks or podcasts.
8. Difficulty following conversations when two or more people are talking.
9. Difficulty hearing alarms or warning signals.
10. Difficulty hearing announcements in airports or train stations.
11. Difficulty hearing at a distance.
12. Difficulty hearing children and women (who typically have higher-pitched voices).
13. Difficulty hearing consonants.
14. Difficulty hearing during medical appointments.
15. Difficulty hearing during physical activities (like exercise, swimming).
16. Difficulty hearing high-pitched sounds (like 's' or 'th').
17. Difficulty hearing in classrooms or lecture halls.
18. Difficulty hearing in public restrooms.
19. Difficulty hearing in religious services or gatherings.
20. Difficulty hearing in shopping centres or crowded places.
21. Difficulty hearing in the car.
22. Difficulty hearing in the workplace.
23. Difficulty hearing low-pitched sounds (like 'v' or 'z').
24. Difficulty hearing on public transportation.
25. Difficulty hearing on the phone.
26. Difficulty hearing speakers who are not facing you directly.
27. Difficulty hearing when back is turned to a speaker.
28. Difficulty hearing when there are multiple noises or sounds happening at once.
29. Difficulty hearing when there is a lot of reverberation.
30. Difficulty hearing your alarm clock or timers.
31. Difficulty hearing your own voice when speaking or singing.
32. Difficulty in hearing at restaurants or cafes.
33. Difficulty keeping up with group conversations.
34. Difficulty localizing where sounds are coming from.
35. Difficulty participating in sports or other activities.
36. Difficulty perceiving own voice or sounds produced.
37. Difficulty understanding speech in the presence of background noise.
38. Difficulty understanding speech, particularly in noisy environments.
39. Difficulty with spatial awareness due to hearing impairment.
40. Dizziness or balance issues.
41. Emotional exhaustion due to constant communication challenges.
42. Experiencing a sensation of fullness in the ear.
43. Fatigue or headaches after prolonged exposure to noise.
44. Fear for personal safety (unable to hear alarms, traffic, etc.).
45. Feeling stressed due to constant concentration to understand speech.
46. Feelings of being ignored or overlooked in conversations.
47. Feelings of embarrassment or frustration.
48. Frequently asking others to speak more slowly, clearly and loudly.
49. Increased dependency on others for communication.
50. Loss of interest in activities once enjoyed.
51. Lowered self-confidence due to hearing difficulties.
52. Missing important details in conversations.
53. Misunderstanding what others say.
54. Muffled hearing.
55. Needing frequent repetition in conversations.
56. Needing to turn up the volume on the television or radio.
57. Reduced ability to relax due to constant effort to hear.
58. Reduced participation in family activities.
59. Sensitivity to loud sounds.
60. Strain in personal relationships due to communication difficulties.
61. Strain or discomfort with using hearing aids or other devices.
62. Struggling with feeling misunderstood by others.
63. Struggling with self-esteem due to hearing loss.
64. Struggling with understanding accents.
65. Tinnitus, or constant ringing or buzzing in the ears.
66. Trouble hearing certain frequencies.
67. Trouble hearing in theatres, concerts, or public events.
68. Trouble hearing lectures or presentations.
69. Trouble hearing over the phone.
70. Trouble hearing sounds of nature.

71. Trouble hearing whispers.
72. Trouble keeping up with fast-paced conversations.
73. Trouble sleeping due to tinnitus or other disturbances.
74. Trouble understanding spoken words.
75. Withdrawal from conversations.

Condition: Heart failure (n = 75)

1. Abdominal bloating
2. Abnormal heart sounds (heart murmurs)
3. Altered bowel habits
4. Anaemia
5. Anxiety
6. Blue or purple colour on lips, hands, or feet (cyanosis)
7. Blurred vision
8. Changes in skin colour or appearance
9. Chest pain
10. Cold and sweaty skin
11. Confusion or disorientation
12. Constipation
13. Decreased ability to perform daily activities
14. Decreased alertness
15. Decreased peripheral vision
16. Decreased sex drive (loss of libido)
17. Decreased urine production
18. Difficulty concentrating
19. Discomfort or pain in the chest (angina)
20. Dizziness or fainting (syncope)
21. Dry mouth or increased thirst
22. Dry skin
23. Erectile dysfunction in men
24. Fatigue even after ample sleep
25. Fluid buildup in the lungs (pulmonary oedema)
26. Fluid retention causing swelling (oedema) in ankles, legs, feet, or abdomen (ascites)
27. Frequent falls due to weakness or dizziness
28. Frequent infections
29. Frequent pneumonia or bronchitis
30. Frequent urination
31. General body weakness
32. Giddiness or vertigo
33. Hoarseness or voice changes
34. Impaired kidney function
35. Inability to lie flat without shortness of breath (orthopnoea)
36. Increased need to urinate at night (nocturia)
37. Intolerance to cold
38. Intolerance to exercise
39. Involuntary weight loss
40. Irregular periods in women
41. Lack of appetite or nausea
42. light-headedness
43. Liver enlargement
44. Loss of consciousness
45. Loss of coordination or balance
46. Loss of muscle mass
47. Memory problems
48. Mood changes or depression
49. Morning headaches
50. Muscle weakness
51. Need to sleep with multiple pillows (orthopnoea)
52. Night sweats
53. Nocturnal urination (nocturia)
54. Numbness or tingling in the limbs
55. Pain or discomfort in the abdomen
56. Palpitations
57. Persistent coughing or wheezing
58. Persistent feeling of being unwell (malaise)
59. Premature aging
60. Pulsating sensation in the neck
61. Rapid breathing (tachypnoea)
62. Rapid or irregular heartbeat
63. Rapid weight gain due to fluid accumulation
64. Reduced exercise tolerance
65. Reduced sensation of taste
66. Reduced sense of smell
67. Restless leg syndrome
68. Shortness of breath (dyspnoea) during activity
69. Shortness of breath while at rest
70. Sleep problems, such as sleep apnoea or insomnia
71. Swelling in the neck veins
72. Tinnitus (ringing in the ears)
73. Tiredness or fatigue
74. Trouble swallowing (dysphagia)
75. Weight loss without trying (cachexia)

Condition: Hyperlipidaemia (n = 75)

1. Bloating or swelling in the abdomen
2. Bluish colour to lips, fingers, and toes
3. Changes in hearing or ringing in the ears
4. Changes in menstruation in women
5. Changes in mood or behaviour
6. Changes in skin colour (pale or bluish coloration)
7. Chest pain (angina)
8. Chronic dry, itchy skin
9. Cold hands or feet
10. Dark urine
11. Decreased libido
12. Depression or anxiety linked to health concerns
13. Difficulty concentrating or remembering (cognitive impairment)
14. Difficulty sleeping or changes in sleep patterns
15. Difficulty swallowing
16. Difficulty walking due to muscle pain or stiffness
17. Digestive issues, such as bloating, constipation, or diarrhoea
18. Discoloration of the skin (often dark, velvety patches in body folds and creases)
19. Dizziness or fainting
20. Dry mouth or increased thirst
21. Dry or red eyes
22. Erectile dysfunction in men
23. Fatigue, especially during physical exertion
24. Feeling of fullness in the abdomen
25. Feeling unusually cold all the time
26. Flushed or red skin
27. Frequent flu-like symptoms
28. Frequent headaches
29. Frequent urination
30. Hair loss on feet and legs
31. Heart palpitations
32. Heavy or uncomfortable feeling in the legs
33. High blood pressure
34. Increased bruising or bleeding
35. Increased irritability or agitation
36. Increased sensitivity to cold
37. Increased susceptibility to infection
38. Insomnia due to anxiety about health
39. Irregular heartbeat (arrhythmia)
40. Jaundice (yellowing of the skin or eyes)
41. Leg cramps when walking or climbing stairs
42. Loss of appetite
43. Muscle aches and pain
44. Nausea
45. Nosebleeds
46. Numbness or cold in legs or arms
47. Numbness or weakness in the limbs
48. Pain in the jaw, neck, upper abdomen, or back
49. Pain in the left arm or shoulder
50. Persistent cough or wheezing
51. Persistent itchiness
52. Persistent or chronic cough
53. Reduced ability to exercise
54. Sensation of heat, burning, or pain in the lower legs (claudication)
55. Sensation of pins and needles in extremities
56. Shiny skin on the legs
57. Shortness of breath
58. Shortness of breath with minimal exertion
59. Slower healing of wounds
60. Sores on feet that do not heal
61. Sudden or unexplained rash or hives
62. Sweating
63. Swelling in the ankles, feet, legs, abdomen, and veins in the neck
64. Tightness or pressure in the chest
65. Unexplained bruises or bleeding
66. Unexplained fatigue or lethargy
67. Unexplained weight loss
68. Unusual hair loss or thinning
69. Unusual or excessive sweating
70. Unusual or persistent stomach pain
71. Unusual or unexpected weight gain
72. Vision problems or changes
73. Weaker pulse in lower limbs
74. Weakness or loss of strength
75. Yellowish patches around the eyes or on the skin (xanthomas

Condition: Hypertension (n = 75)

1. Abdominal pain
2. Anxiety
3. Blood in the urine
4. Blood spots in the eyes
5. Blurred vision
6. Bone pain
7. Change in sense of smell
8. Changes in heart rhythm
9. Changes in skin colour
10. Changes in speech
11. Chest pain
12. Chest tightness
13. Clammy skin
14. Cold feet and hands
15. Concentration difficulties
16. Confusion
17. Constant thirst
18. Dark urine
19. Decreased sexual desire
20. Decreased urine output
21. Depression
22. Difficulty moving arms or legs
23. Difficulty seeing in one or both eyes
24. Difficulty sleeping
25. Difficulty swallowing
26. Dizziness
27. Dry mouth
28. Early morning headaches
29. Erectile dysfunction in men
30. Excessive sleepiness
31. Fainting
32. Fatigue
33. Feeling unusually cold all the time
34. Flushing of the face
35. Frequent falls
36. Frequent illnesses or infections
37. Frequent nosebleeds
38. Frequent urination
39. General weakness
40. Headaches
41. Increased sensitivity to cold or heat
42. Irregular periods in women
43. Loss of balance
44. Loss of consciousness
45. Memory loss
46. Metallic taste
47. Mood changes
48. Nausea
49. Nosebleeds
50. Nosebleeds not related to other health issues
51. Numbness or tingling in hands or feet
52. Palpitations (irregular heartbeats)
53. Persistent cough
54. Persistent hiccups
55. Persistent itchiness
56. Rapid heartbeat
57. Reduced exercise tolerance
58. Shortness of breath
59. Sleeping problems
60. Stress
61. Sweating
62. Swelling in the neck (possible sign of kidney problems)
63. Swelling of the hands
64. Swollen ankles, feet or legs (oedema)
65. Tinnitus (ringing in the ears)
66. Tremors
67. Unexplained muscle aches
68. Unexplained weight loss or gain
69. Unsteady walking
70. Unusual bruising
71. Unusual hair loss or growth
72. Unusual hunger or lack of appetite
73. Unusual sweating
74. Vertigo (sense of spinning)
75. Yellowish colour of the skin or eyes (jaundice)

Condition: Hypothyroidism (n = 75)

1. Abnormal sweating
2. Anaemia
3. Anxiety
4. Blood clots
5. Brittle nails
6. Carpal tunnel syndrome
7. Changes in bowel habits
8. Changes in personality or behaviour
9. Changes in vision
10. Constipation (Hypothyroidism)
11. Decreased appetite (Hypothyroidism)
12. Decreased libido
13. Decreased taste and smell
14. Delayed puberty in teenagers
15. Depression
16. Diarrhoea (Hyperthyroidism)
17. Difficulty getting pregnant
18. Difficulty regulating body temperature
19. Difficulty sleeping
20. Difficulty swallowing
21. Dizziness
22. Dry skin and hair (Hypothyroidism)
23. Erectile dysfunction
24. Eye irritation and tearing
25. Fainting
26. Fatigue
27. Frequent urination
28. Goitre (swelling in the neck from an enlarged thyroid gland)
29. Hair loss
30. Hand tremors (Hyperthyroidism)
31. Headaches
32. Hearing loss
33. High blood pressure
34. High cholesterol (Hypothyroidism)
35. Hoarse voice
36. Increased appetite (Hyperthyroidism)
37. Increased sensitivity to medications
38. Increased susceptibility to infections
39. Increased thirst
40. Infertility
41. Irregular menstrual periods
42. Irritability
43. Itchiness
44. Joint pain
45. Loss of interest in hobbies
46. Low blood pressure
47. Memory problems
48. Miscarriage or premature birth in pregnant women
49. Mood swings
50. Muscle aches
51. Muscle weakness
52. Nausea
53. Obsessive-compulsive symptoms
54. Pale or yellowish skin
55. Panic disorder
56. Poor concentration
57. Protruding eyeballs (Hyperthyroidism)
58. Puffy face (Hypothyroidism)
59. Rapid heart rate (Hyperthyroidism)
60. Ringing in the ears
61. Sensitivity to cold (Hypothyroidism)
62. Sensitivity to heat (Hyperthyroidism)
63. Shortness of breath
64. Skin rashes (Hyperthyroidism)
65. Sleep apnoea
66. Slow growth in children
67. Slow heart rate (Hypothyroidism)
68. Slow wound healing
69. Slowed mental functioning (Hypothyroidism)
70. Swelling in the joints
71. Swollen ankles
72. Tinnitus
73. Vomiting
74. Weight gain (Hypothyroidism)
75. Weight loss (Hyperthyroidism)

Condition: Kidney Disease (n = 75)

1. Acid reflux
2. Anaemia
3. Anxiety
4. Bad breath
5. Blood in urine (haematuria)
6. Bone fractures
7. Bone pain
8. Bruising easily
9. Changes in skin colour
10. Changes in taste or mouth sores
11. Changes in urine colour
12. Chest pain
13. Cold intolerance
14. Coma
15. Decreased appetite
16. Decreased mental sharpness
17. Decreased sex drive
18. Depression
19. Difficulty concentrating
20. Difficulty in regulating body temperature
21. Dizziness
22. Dry skin
23. Enlarged heart (cardiomegaly)
24. Erectile dysfunction
25. Fatigue or weakness
26. Feelings of malaise
27. Fluid in the lungs (pulmonary oedema)
28. Fluid retention (oedema)
29. Foamy urine
30. Frequent hiccups
31. Frequent urination
32. Gastrointestinal bleeding
33. Headaches
34. Hearing problems
35. Heart palpitations
36. High blood pressure (hypertension)
37. Hyperkalaemia (high potassium levels in blood)
38. Hyperphosphatemia (high phosphorus levels in blood)
39. Hypertension that's difficult to control
40. Hypocalcaemia (low calcium levels in blood)
41. Inability to urinate
42. Increased susceptibility to infections
43. Increased sweating
44. Infertility
45. Insomnia
46. Irregular heartbeat
47. Less frequent urination
48. Loss of libido
49. Lower back pain
50. Memory problems
51. Menstrual irregularities
52. Muscle cramps
53. Nausea
54. Nighttime urination (nocturia)
55. Nosebleeds
56. Pale skin
57. Peripheral neuropathy
58. Persistent itching
59. Poor growth (in children)
60. Protein in urine (proteinuria)
61. Puffiness around the eyes
62. Reduced muscle mass
63. Restless legs syndrome
64. Seizures
65. Shortness of breath
66. Skin rashes/itching
67. Sleep problems
68. Stunted growth in children
69. Swelling of feet and ankles
70. Thinning hair
71. Trouble sleeping
72. Urinary urgency
73. Vomiting
74. Weight loss
75. Yellow skin (jaundice)

Condition: Liver Disease (n = 75)

1. Abdominal bloating
2. Abdominal pain
3. Anxiety
4. Ascites (abdominal swelling due to fluid buildup)
5. Bitter taste in the mouth
6. Bone loss (osteoporosis)
7. Change in body temperature regulation
8. Changes in handwriting
9. Changes in sense of smell
10. Changes in sense of taste
11. Changes in skin colour
12. Changes in sleep patterns
13. Chronic fatigue
14. Cognitive impairment
15. Dark urine
16. Decreased urine output
17. Depression
18. Difficulty concentrating
19. Digestive issues
20. Dizziness
21. Dry eyes
22. Dry mouth
23. Easy bruising or bleeding
24. Enlarged liver (hepatomegaly)
25. Enlarged spleen (splenomegaly)
26. Fatigue
27. Fever
28. Fluid retention
29. Foul breath
30. Gallstones
31. Gynecomastia (breast enlargement in males)
32. Hair loss
33. Hallucinations
34. Hepatic encephalopathy (confusion, disorientation)
35. Hepatorenal syndrome (kidney failure)
36. Hyperpigmentation of the skin
37. Impotence
38. Increased sensitivity to alcohol and drugs
39. Increased susceptibility to infections
40. Insulin resistance/diabetes
41. Itching (pruritus)
42. Jaundice (yellowing of the skin and eyes)
43. Loss of appetite
44. Loss of sex drive (libido)
45. Low-grade fever
46. Memory loss
47. Menstrual irregularities
48. Metallic taste in mouth
49. Mood changes
50. Muscle or joint pain
51. Muscle weakness
52. Nail changes
53. Nausea
54. Night sweats
55. Pale stool
56. Peripheral neuropathy
57. Personality changes
58. Portal hypertension (high blood pressure in the liver's blood vessels)
59. Reduced muscle mass
60. Restless leg syndrome
61. Right shoulder pain
62. Sensitivity to light (photophobia)
63. Sensitivity to medications
64. Shortness of breath
65. Skin rashes
66. Sleep disturbances
67. Slurred speech
68. Spider angiomas (small, spider-like blood vessels on the skin)
69. Stools with a strong odour
70. Swelling in the legs (oedema)
71. Symptoms of anaemia like weakness or fatigue
72. Tremors
73. Unexplained weight loss
74. Varices (swollen veins in the oesophagus or stomach that can bleed)
75. Vomiting

Condition: Musculoskeletal conditions (including back pain) (n = 75)

1. Acute pain
2. Back pain that improves when lying down
3. Bone fractures from minor trauma
4. Bone pain or tenderness
5. Burning sensation
6. Chronic pain
7. Clicking or cracking sound in joints
8. Constant pain
9. Cramps
10. Crepitus (grating sensation in the joints)
11. Deformities of the joints
12. Difficulty bending or lifting
13. Difficulty climbing stairs
14. Difficulty gripping objects
15. Difficulty raising arms above the head
16. Difficulty sitting or standing for long periods
17. Difficulty sleeping due to pain
18. Difficulty turning the head side to side
19. Difficulty walking
20. Difficulty with balance
21. Difficulty with daily activities (ADLs)
22. Disturbed sleep patterns
23. Dull ache
24. Feeling of bones grating against each other
25. Foot drop (inability to lift the front part of the foot)
26. Frequent falls or clumsiness
27. Gradual onset of pain
28. Heaviness in limbs
29. Increased pain during physical activity
30. Inflammation
31. Intermittent pain
32. Involuntary muscle movements
33. Joint stiffness
34. Limited range of motion
35. Locking of the joint
36. Loss of appetite due to pain
37. Loss of bowel or bladder control (medical emergency)
38. Loss of height due to vertebral compression fractures
39. Loss of muscle coordination
40. Morning stiffness
41. Muscle atrophy
42. Muscle fatigue
43. Muscle spasms
44. Muscle weakness
45. Night sweats
46. Numbness
47. Pain in the knees
48. Pain in the neck
49. Pain in the shoulders
50. Pain radiating down the leg
51. Pain worsening in cold weather
52. Pain worsening in hot weather
53. Pain worsening with specific movements
54. Persistent fatigue
55. Pins and needles sensation
56. Postural changes
57. Psychological distress (depression/anxiety due to chronic pain)
58. Redness over the affected area
59. Reduced flexibility
60. Reduced overall mobility
61. Reduced physical endurance
62. Relieved pain at rest
63. Sciatica (pain along the sciatic nerve)
64. Sensitivity to changes in weather
65. Sharp pain
66. Shooting or stabbing pain
67. Sudden onset of pain
68. Swelling
69. Tenderness to the touch
70. Tingling sensation
71. Twinges
72. Unexplained bruising
73. Unexplained fevers (may be indicative of infection or an inflammatory condition)
74. Unintentional weight loss
75. Waddling gait

Condition: Obesity (n = 75)

1. Acanthosis nigricans (dark, thick skin in certain areas)
2. Acid reflux/GERD
3. Anxiety
4. Asthma
5. Back pain
6. Blood clots
7. Body image distress
8. Cardiovascular disease
9. Certain cancers (e.g., breast, colon)
10. Chronic kidney disease
11. Complications from surgery
12. Coronary artery disease
13. Daytime sleepiness or fatigue
14. Decreased stamina
15. Depression
16. Difficulty in finding clothing and accessories
17. Difficulty sleeping
18. Difficulty with certain medical imaging (due to weight restrictions)
19. Difficulty with temperature regulation
20. Difficulty with wound healing
21. Erectile dysfunction
22. Excess body fat
23. Fatigue or difficulty with physical exertion
24. Fatty liver disease
25. Gallbladder disease
26. Gout
27. Heart disease
28. High blood pressure
29. High body mass index (BMI)
30. High cholesterol
31. Hypertension
32. Impaired balance
33. Impaired immune function
34. Impaired social life
35. Increased healthcare costs
36. Increased hunger
37. Increased pain sensitivity
38. Increased risk of dementia
39. Increased risk of falls
40. Increased risk of infections
41. Increased surgical risk
42. Increased sweating
43. Infertility
44. Inflammation
45. Insulin resistance
46. Irregular menstrual cycles
47. Joint pain
48. Liver disease
49. Metabolic syndrome
50. Mobility issues
51. Mobility issues related to public transportation
52. Occupational disability
53. Osteoarthritis
54. Peripheral artery disease
55. Polycystic ovary syndrome (PCOS)
56. Pregnancy complications
57. Psychological distress/stigmatization
58. Rashes or infections in skin folds
59. Reduced access to certain healthcare options due to weight restrictions
60. Reduced libido
61. Reduced life expectancy
62. Reduced personal and work productivity
63. Reduced quality of life
64. Reduced self-esteem
65. Respiratory problems
66. Shortness of breath
67. Skin tags
68. Sleep apnoea
69. Sleep disorders
70. Snoring
71. Social isolation
72. Stroke
73. Type 2 diabetes
74. Urinary incontinence
75. Varicose veins

Condition: Osteoporosis (n = 75)

1. Painful fractures
2. Bone pain or tenderness
3. Back pain, due to fractures or collapsed vertebra
4. Loss of height over time
5. Stooped posture (Dowager's hump)
6. Reduced mobility
7. Reduced ability to carry out daily activities
8. Decreased lung capacity (due to compressed vertebrae)
9. Difficulty sleeping due to pain
10. Anxiety about potential fractures
11. Depression due to reduced mobility or chronic pain
12. Difficulty bending and stooping
13. Increased risk of falling
14. Tooth loss (due to lower bone density in the jaw)
15. Fracture that occurs more easily than expected
16. Reduced physical fitness
17. Impaired balance
18. Difficulty with personal care activities due to fractures
19. Negative impact on body image (due to changes in posture)
20. Chronic pain
21. Reduced independence
22. Social isolation
23. Lower overall quality of life
24. Fear of engaging in social activities due to risk of fracture
25. Decreased productivity at work
26. Financial burden of treating and managing the disease
27. Side effects of medications
28. Strained personal relationships due to chronic pain or disability
29. Stress and anxiety about the future
30. Weight loss due to lack of appetite or difficulty preparing meals
31. Digestive problems if diet changes (due to dietary calcium and Vitamin D recommendations)
32. Side effects of calcium supplements, like constipation
33. Difficulty traveling
34. Changes in sexual health and relationships
35. Difficulty with bending, lifting, or twisting
36. Fear of dependency
37. Fear of being a burden on family or caregivers
38. Concern about potential nursing home placement
39. Difficulty participating in recreational activities
40. Altered nutritional status due to changes in dietary habits
41. Necessity to use assistive devices (like canes or wheelchairs)
42. Difficulty driving
43. Decreased ability to exercise
44. Fear of exercising due to potential fractures
45. Difficulty dressing oneself
46. Difficulty managing personal hygiene
47. Difficulty maintaining a clean and organized living space
48. Emotional distress
49. Increased risk of complications from surgeries
50. Increased risk of hospitalization
51. Difficulty participating in community events
52. Challenges adapting to lifestyle modifications
53. Increased risk of other chronic diseases due to lack of mobility
54. Changes in self-esteem
55. Increased susceptibility to scams or abuse due to perceived vulnerability
56. Decreased ability to cope with stress
57. Potential for drug interactions due to multiple medications
58. Necessity to regularly visit healthcare providers for monitoring
59. Difficulty managing comorbid health conditions
60. Increased risk of bedsores due to immobility
61. Risk of complications like pneumonia or deep vein thrombosis after fractures
62. Increased difficulty in managing other aspects of personal health (such as weight, blood pressure, diabetes)
63. Difficulty using public transportation
64. The need to modify the home for safety (like installing handrails)
65. Difficulty walking on uneven surfaces
66. Fear of weather conditions that might increase the risk of falls (like ice or snow)
67. The need for physical rehabilitation or physical therapy
68. Difficulty maintaining a regular sleep pattern due to pain or anxiety
69. Changes in social roles and relationships
70. Potential for increased alcohol or substance use to manage chronic pain

71. Difficulty adapting to new physical limitations
72. Difficulty maintaining a regular work schedule due to health appointments or decreased physical abilities
73. Difficulty with stairs
74. Difficulty caring for dependents (such as children or aging parents)
75. Grief over the loss of the previous lifestyle

Condition: Stroke (n = 75)

1. Agitation or restlessness
2. Anxiety or panic attacks
3. Apathy or lack of motivation
4. Behavioural changes, including irritability or lack of self-awareness
5. Bowel or bladder incontinence
6. Changes in appetite
7. Changes in personality
8. Depression or feelings of sadness
9. Difficulties in multitasking
10. Difficulties with gross motor skills
11. Difficulty adjusting to changes in routine
12. Difficulty calculating or working with numbers
13. Difficulty controlling emotions
14. Difficulty focusing or paying attention
15. Difficulty identifying objects by touch (stereognosis)
16. Difficulty interpreting facial expressions or social cues
17. Difficulty interpreting touch (astereognosis)
18. Difficulty perceiving differences in temperature
19. Difficulty performing daily tasks (like bathing, dressing, or eating)
20. Difficulty reading or writing
21. Difficulty recognizing familiar people or objects (agnosia)
22. Difficulty recognizing own reflection (prosopagnosia)
23. Difficulty recognizing sounds (auditory agnosia)
24. Difficulty swallowing (dysphagia)
25. Difficulty understanding written words (alexia)
26. Difficulty with fine motor skills
27. Disorientation to time or place
28. Dizziness, loss of balance, or unexplained falls
29. Emotional outbursts or emotional instability
30. Excessive crying or laughing
31. Excessive sweating
32. Fear of being alone
33. Feeling of "pins and needles" or tingling sensation
34. Hallucinations or delusions
35. Hearing loss or tinnitus
36. Impaired judgment or decision making
37. Impaired reasoning abilities
38. Impulsivity or risk-taking behaviour
39. Inability to carry out a sequence of movements (ideational apraxia)
40. Inability to carry out learned purposeful movements (apraxia)
41. Inability to follow or understand a conversation
42. Inability to plan and organize
43. Inability to recognize one's own body parts (autotopagnosia)
44. Increased sensitivity to pain
45. Increased tone in muscles leading to stiffness (spasticity)
46. Involuntary eye movements
47. Lack of empathy or understanding of others' emotions
48. Lack of spatial awareness
49. Loss of temperature sensation
50. Loss of visual field (hemianopsia)
51. Memory loss
52. Mood swings
53. Neglecting one side of the body (neglect syndrome)
54. Paralysis (often on one side of the body)
55. Poor coordination or clumsiness
56. Problems with visual perception
57. Rapid, uncontrollable eye blinking (blepharospasm)
58. Severe, sudden headache with no known cause
59. Sexual dysfunction
60. Slurred speech (dysarthria)
61. Sudden confusion or trouble understanding speech
62. Sudden numbness or weakness in the face, arm, or leg (especially on one side of the body)
63. Sudden vision problems in one or both eyes
64. Swallowing difficulties leading to aspiration pneumonia
65. Temporary loss of consciousness
66. Trouble controlling movements or walking (ataxia)
67. Trouble sleeping or insomnia
68. Trouble speaking or articulating thoughts
69. Trouble with spatial relationships (topographical disorientation)
70. Uncontrollable movements (chorea)
71. Unexplained weight loss or gain
72. Unusual or unexplained fatigue
73. Unusual sense of taste or smell
74. Vertigo
75. Visual spatial difficulties

Condition: Urinary incontinence (n = 75)

1. Anxiety about urination in public places or when away from home.
2. Avoidance of sexual activities.
3. Avoidance of social activities.
4. Back pain.
5. Bladder stones: Hard masses of minerals in your bladder.
6. Changes in bowel habits.
7. Changes in the colour of urine.
8. Changes in the smell of urine.
9. Decreased force in the urine stream.
10. Decreased productivity at work.
11. Decreased self-esteem.
12. Decreased sexual desire.
13. Dehydration (if avoiding liquids to control urination).
14. Delayed recognition or diagnosis of condition.
15. Dependence on medications or surgical treatments.
16. Depression or irritability due to chronic symptoms.
17. Difficulty participating in physical activities due to worry about urine leakage.
18. Difficulty with spontaneity.
19. Discomfort or pain when sitting.
20. Disrupted travel plans.
21. Distress due to frequent doctor or hospital visits.
22. Dribbling at the end of urination.
23. Dysuria: Painful or difficult urination.
24. Embarrassment or shame.
25. Erectile dysfunction.
26. Fatigue due to disrupted sleep.
27. Fear of complications or progression of the disease.
28. Fear of stigmatization.
29. Feelings of frustration or anger.
30. Financial strain due to treatment costs.
31. Foul-smelling urine.
32. Frequent need to change clothes due to urine leakage.
33. Frequent urination: Needing to urinate more often than usual.
34. Frustration over lack of control over symptoms.
35. Groin pain.
36. Haematuria: Blood in the urine.
37. Impact on family planning decisions.
38. Incomplete bladder emptying: The feeling of still having urine in the bladder after urination.
39. Increased abdominal size.
40. Increased health care costs.
41. Increased need to use pads or diapers.
42. Increased strain in personal relationships.
43. Infertility (in rare cases).
44. Interference with leisure activities.
45. Interference with romantic relationships.
46. Interrupted sleep.
47. Kidney damage (in severe cases of urinary retention).
48. Lower abdominal pain or discomfort.
49. Lower backache.
50. Lower quality of life.
51. Need for frequent medical follow-up visits.
52. Negative impact on body image.
53. Nocturia: Needing to wake up to urinate multiple times during the night.
54. Overflow incontinence: Inability to completely empty the bladder, leading to frequent or constant dribbling of urine.
55. Painful ejaculation.
56. Pelvic floor muscle weakness.
57. Pelvic pain.
58. Recurrent urinary tract infections.
59. Reduced ejaculation force.
60. Reduced participation in physical activities.
61. Sensation of pressure in the bladder.
62. Skin irritation and rashes due to urinary incontinence.
63. Social isolation due to worry about urine leakage.
64. Stigma associated with urinary problems.
65. Straining to urinate.
66. Stress incontinence: Leakage of urine when pressure is exerted on the bladder by coughing, sneezing, laughing, exercising or lifting something heavy.
67. Swelling in the lower extremities (in severe cases of urinary retention).
68. Trouble concentrating due to frequent need to urinate.
69. Urinary hesitancy: Difficulty starting urination.
70. Urinary incontinence: Involuntary leakage of urine.

71. Urinary tract infections: Frequent infections due to urinary retention.
72. Urinary urgency: The sudden urge to urinate due to involuntary contractions of the bladder muscles.
73. Urine leakage during sexual activity.
74. Weak urine flow: Urine stream is slow or comes out in dribbles.
75. Weight gain (due to avoidance of physical activity).

Condition: Visual impairment (n = 75)

1. A growth or mole on the eye or eyelid
2. Appearance of a dark curtain shading your vision
3. Blood in the white part of the eye
4. Blurred vision
5. Bulging of one or both eyes
6. Changes in how you see texture or detail
7. Changes in the colour of the iris
8. Changes in the way you see colour (Colour blindness)
9. Cloudy vision
10. Colours appearing faded or washed out
11. Constant eye rubbing
12. Crossed eyes, or eyes that appear to move independently of each other (Strabismus)
13. Dark spots in the centre of your field of vision
14. Difficulty adjusting from dark to light environments
15. Difficulty focusing on close or distant objects
16. Difficulty reading or recognizing faces
17. Difficulty seeing at night (Nyctalopia)
18. Difficulty seeing in dim light
19. Difficulty seeing things out of the corner of your eye
20. Difficulty tracking moving objects with your eyes
21. Difficulty with depth perception
22. Discharge from the eyes
23. Distorted vision (straight lines appearing wavy)
24. Double vision
25. Dry eyes
26. Dull or sharp eye pain
27. Excessive tearing or watery eyes
28. Eye fatigue or strain after short periods of reading or computer use
29. Eye that turns inward or outward
30. Eyes that don't point in the same direction at the same time
31. Feeling like there's something in the eye (foreign body sensation)
32. Flashes of light or dark spots in vision
33. Flashing lights
34. Flickering or shimmering vision
35. Floating specks or strings in vision
36. Frequent changes in glasses or contact lens prescription
37. Frequent headaches
38. Frequent squinting
39. Inability to close the eye
40. Inability to maintain eye contact
41. Inability to open the eye
42. Inability to see at certain distances (Myopia, Hyperopia)
43. Inability to see certain colours
44. Involuntary eye twitching
45. Itchy eyes
46. Loss of peripheral (side) vision
47. Misaligned eyes
48. Needing more light to see or read
49. Pain in the eye
50. Partial vision loss
51. Poor visual acuity
52. Problems with glare
53. Problems with peripheral vision
54. Rapid eye movement
55. Redness in the eyes
56. Seeing a blank spot in your field of vision
57. Seeing halos around lights
58. Seeing images at a distorted scale (Micropsia or Macropsia)
59. Seeing light flashes, even when your eyes are closed
60. Seeing multiple images
61. Seeing rainbows or halos around light
62. Seeing things that aren't there (Visual hallucinations)
63. Seeing zigzag lines
64. Sensitivity to light (Photophobia)
65. Slow central vision loss
66. Spots or floaters in the field of vision
67. Sudden loss of vision
68. Swollen, red eyelids
69. Temporary blindness in one eye
70. Tunnel vision (narrow field of vision)
71. Unequal pupil sizes
72. Unusual eye movements
73. Vision loss in one part of your field of vision
74. Vision loss that comes and goes
75. Vision that seems to pulsate

Appendix 6: Jaccard indices as a measure of list similarity comparing symptoms extracted from condition-specific PROMs and ChatGPT

Condition	Symptoms ePROVIDE (n)	Symptoms (ChatGPT) (n)	Shared items	Coverage ePROVIDE by ChatGPT (%)	Coverage ChatGPT (%) by ePROVIDE	Jaccard Index
Alcohol Misuse	0	70	0	0	0	0.00
Angina	0	773	0	0	0	0.00
Arthritis	36	51	4	11.11	7.84	0.05
Asthma	0	64	0	0	0	0.00
Cancer	191	73	32	16.75	43.8	0.14
Crohn's Disease	0	66	0	0	0	0.00
Dementia	4	69	2	50.0	2.9	0.03
Depression	43	71	13	30.23	18.31	0.13
Diabetes	29	61	10	34.48	16.39	0.13
Eczema	0	59	0	0	0	0.00
Gastro-oesophageal reflux disease	50	57	15	30.00	26.32	0.16
Hearing Loss	0	41	0	0	0	0.00
Heart Failure	18	73	7	38.89	9.59	0.08
Hyperlipidaemia	0	73	0	0	0	0.00
Hypertension	61	70	24	39.34	34.29	0.22
Hyperthyroidism	0	60	0	0	0	0.00
Kidney Disease	12	58	8	66.67	13.8	0.13
Liver Disease	29	60	9	31.03	15.0	0.11
Musculoskeletal	54	69	8	14.81	11.59	0.07
Obesity	0	39	0	0	0	0.00
Osteoporosis	4	25	2	50.00	8.00	0.07
Stroke	0	68	0	0	0	0.00
Urinary Incontinence	1	57	1	100.00	1.8	0.02
Visual Impairment	29	57	2	6.90	3.51	0.02

Appendix 7: Summary of pooled PROM and ChatGPT symptom list screening with reasons for symptom exclusion. Symptoms are organised according to the conceptual domains of the SBQ™-LC.

			Reasons for exclusion						
**SBQ domain**	**Symptoms for clinical review**	**Symptoms excluded**	**Not clinically relevant to MLTC[a]**	**Redundant[b]**	**Vague[c]**	**Not suitable for patient-report[d]**	**Multiple concepts[e]**	**Too specific[f]**	**Total included symptoms**
Breathing	21	12	2	5	5	0	0	0	9
Circulation	44	32	5	23	1	3	0	0	12
ENT	114	105	15	89	1	0	0	0	9
Eyes	99	84	35	40	5	4	0	0	15
Fatigue	40	35	3	27	3	2	0	0	5
Impact on Daily Life	181	171	64	63	26	3	3	12	10
Memory, Thinking, Communication	156	140	27	74	23	16	0	0	16
Sexual Health	37	32	11	14	7	0	0	0	5
Movement	64	56	23	12	9	5	3	4	8
Mental Health/Wellbeing	233	211	71	68	37	11	0	24	22
Muscles and Joints	116	103	31	42	20	10	0	0	13
Pain	75	66	19	23	19	5	0	0	9
Skin and Hair	101	97	35	27	15	14	3	3	4
Sleep	50	45	2	18	18	0	1	6	5
Stomach & Digestion	118	101	17	63	9	1	3	8	17
Substance Use*	7	6	0	0	1	5	0	0	1
Other Symptoms	152	123	22	72	7	17	2	3	29
Unallocated symptoms**	24	23	5	5	4	9	0	0	1
**TOTAL**	**1632**	**1442**	387	665	210	105	15	60	**190**

*Not a domain of the SBQ™-LC; **Unallocated symptoms: Symptoms not included in the source instrument's (SBQ-LC) conceptual framework; [a]Not clinically relevant to MLTC: Symptom not prevalent nor informative for clinical management; [b]Redundant: duplicate/semantically redundant with another item/concept; [c]Vague: meaning is ambiguous; [d]Not suitable for patient-report: item content was a clinical sign or otherwise unsuitable for self-report; [e]Multiple concepts: two or more symptom concepts within a single item making it unclear which concept the respondent is answering; [f]Too specific: symptom concept is specific to a single condition, reducing its general applicability. Definitions based on DeWalt, D. A., Rothrock, N., Yount, S., & Stone, A. A. (2007). Evaluation of item candidates: The PROMIS qualitative item review. *Medical Care*, 45(5 SUPPL. 1), 12–21. https://doi.org/10.1097/01.mlr.0000254567.79743.e2.

Appendix 8: Clinical specialities represented in the HCP sample (N = 17)

Speciality	N*
Cardiology	1
Dentistry	1
Dermatology	1
Gastroenterology	1
General practice	4
Geriatric medicine	1
Obstetrics and gynaecology	2
Ophthalmology	1
Otolaryngology	1
Psychiatry	1
Psychology and mental health	2
Respiratory medicine	2
Rheumatology	2
Sleep	2
Urology	1

*Total exceeds n = 17 due to HCP expertise across multiple specialities.

Appendix 9: Summary of HCP review and symptom shortlisting for each domain of the SBQ-LC (source instrument) conceptual framework

Domain	Symptoms reviewed by HCPs	Symptoms included⁺
Breathing	9	9
Circulation	12	8
Ear, Nose and Throat	9	5
Eyes	15	11
Fatigue	5	4
Impact on Daily Life	10	8
Memory, thinking and communication	16	8
Mental Health and Wellbeing	22	18
Movement	8	8
Muscles and Joints	13	14
Oral Health*	0	8
Pain	9	1
Sexual and Reproductive Health	5	4
Skin and Hair	4	4
Sleep	5	5
Stomach and Digestion	17	14
Substance Use*	1	0
Urinary Symptoms*	0	8
Other Symptoms	30	14
**TOTAL**	**190**	**151**

*Not a domain of the source instrument (SBQ™-LC)

⁺For some domains, number of included items > original number of symptoms due to reassignment of item from another domain.

Appendix 10: Symptoms identified during concept elicitation

Domain	Symptom concepts identified*
Breathing	Chest tightness, inability to lie flat without shortness of breath, short of breath climbing stairs, short of breath at rest, short of breath walking, wheezing, cough, coughing up blood, excess mucus production
Circulation	Intolerance to cold, sensation of heat, burning, pain in lower leg, dizziness, fainting, heart palpitations, light-headedness, swelling of ankles/legs, chest pain
Ear, nose and throat	Blocked or runny nose, hearing loss, hoarseness or voice changes, tinnitus, vertigo
Eyes	Blurred vision, change in way see colour, difficulty noticing object at the side, difficulty seeing at night, double vision, dry eyes, inability to close eye(s), partial vision loss, problems with glare, seeing blank spot in field of vision, seeing haloes around lights
Fatigue	Feeling weak, fatigue, lack of energy, tiredness
Memory, thinking and communication	Difficulty with speech (articulation), Brain fog, concentration difficulties, Word finding difficulty, difficulty identifying faces, difficulty understanding speech, strain in personal relationships due to communication difficulties, trouble concentrating on conversation
Mental health and wellbeing	Restlessness/wound up/on edge, anxiety, anxiety or panic attacks, decreased self-esteem, depression, feeling like a burden, feeling worthless/excessive guilt, increased appetite, isolation/withdrawal from social activities, lack of interest in hobbies, lack of motivation, mentally exhausted, stress, craving alcohol/drugs, felt unwell if not using alcohol/drugs, worried about substance use, self-harm, suicidal thoughts
Movement	Difficulty gripping objects, balance problems, difficulty climbing stairs, difficulty raising arms above head, difficulty standing, difficulty walking, require a walking aid/assistance, hand tremors
Muscles and joints	"Pins and needles" sensation, frequent falls, joint pain, joint stiffness, muscle aches, joint swelling, difficulty rising from sitting, weakness one-side of body, weakness/loss of strength, back pain, discomfort hip/groin, discomfort neck, shoulder pain, pain radiating down leg
Oral health	Bleeding gums, bad breath, tooth loss, dental erosions/cavities, dry mouth, mouth ulcers, sore mouth, swollen gums

Do Not Copy

Domain	Symptom concepts identified*
Pain	Pain
Sexual and reproductive health	Abnormal bleeding from vagina (female), decreased libido (female), decreased libido (male), erectile dysfunction
Skin and hair	Sores, ulcers or infections that won't heal, dry/itchy skin, rash, unusual hair loss/thinning
Sleep	Excessive sleep, difficulty falling asleep, frequent waking, early-morning waking, daytime sleepiness or fatigue
Stomach and digestion	Feeling excessively full, Diarrhoea, bloating, abdominal pain, reflux, altered bowl habits, blood in stools, constipation, dark tarry stools, incontinence, mucus in stool, nausea, vomiting blood, difficulty swallowing
Urinary symptoms	Bood in urine, decreased urine output, difficulty urinating, frequent urination, urinary incontinence, urgency, frequent night time urination, feeling of incomplete bladder emptying
Other symptoms	Cold, thirst, headache, fever, heat intolerance, change in taste, bruising, night sweats, excessive sweating, weight gain, seizure, appetite, weight loss, swelling in neck
Impact on daily life	Bothered by treatment side effects, feeling ill, difficulty maintaining personal relationships, difficulty shopping, difficulty with light housework, impaired social life, limited to how long one can work, help with self-care

***Symptom concepts do not represent the final list of concepts, final item wording, or instrument structure which will be determined in subsequent stages of instrument development and validation.**

Do Not Copy
